# Sex-Specific Cross Tissue Meta-Analysis Identifies Immune Dysregulation in Women With Alzheimer’s Disease

**DOI:** 10.3389/fnagi.2021.735611

**Published:** 2021-09-30

**Authors:** Manish D. Paranjpe, Stella Belonwu, Jason K. Wang, Tomiko Oskotsky, Aarzu Gupta, Alice Taubes, Kelly A. Zalocusky, Ishan Paranjpe, Benjamin S. Glicksberg, Yadong Huang, Marina Sirota

**Affiliations:** ^1^Bakar Computational Health Sciences Institute, University of California, San Francisco, San Francisco, CA, United States; ^2^Harvard-MIT Program in Health Sciences and Technology, Harvard Medical School, Boston, MA, United States; ^3^Pharmaceutical Sciences and Pharmacogenomics Graduate Program, University of California, San Francisco, San Francisco, CA, United States; ^4^Department of Pediatrics, University of California, San Francisco, San Francisco, CA, United States; ^5^The Gladstone Institute of Neurological Disease, San Francisco, CA, United States; ^6^Department of Neurology, University of California, San Francisco, San Francisco, CA, United States; ^7^Hasso Plattner Institute for Digital Health at Mount Sinai, Icahn School of Medicine at Mount Sinai, New York, NY, United States

**Keywords:** sex, transcriptomics, biomarker, APOE, inflammation, neuroinflammation, Alzheimer’s disease

## Abstract

**Background:** Alzheimer’s disease (AD) is a progressive neurodegenerative disorder and the most common cause of dementia in the United States. In spite of evidence of females having a greater lifetime risk of developing Alzheimer’s Disease (AD) and greater apolipoprotein E4-related (APOE ε4) AD risk compared to males, molecular signatures underlying these differences remain elusive.

**Methods:** We took a meta-analysis approach to study gene expression in the brains of 1,084 AD patients and age-matched controls and whole blood from 645 AD patients and age-matched controls in seven independent datasets. Sex-specific gene expression patterns were investigated through use of gene-based, pathway-based and network-based approaches. The ability of a sex-specific AD gene expression signature to distinguish Alzheimer’s disease from healthy controls was assessed using a linear support vector machine model. Cell type deconvolution from whole blood gene expression data was performed to identify differentially regulated cells in males and females with AD.

**Results:** Strikingly gene-expression, network-based analysis and cell type deconvolution approaches revealed a consistent immune signature in the brain and blood of female AD patients that was absent in males. In females, network-based analysis revealed a coordinated program of gene expression involving several zinc finger nuclease genes related to Herpes simplex viral infection whose expression was modulated by the presence of the APOE ε4 allele. Interestingly, this gene expression program was missing in the brains of male AD patients. Cell type deconvolution identified an increase in neutrophils and naïve B cells and a decrease in M2 macrophages, memory B cells, and CD8+ T cells in AD samples compared to controls in females. Interestingly, among males with AD, no significant differences in immune cell proportions compared to controls were observed. Machine learning-based classification of AD using gene expression from whole blood in addition to clinical features produced an improvement in classification accuracy upon stratifying by sex, achieving an AUROC of 0.91 for females and 0.80 for males.

**Conclusion:** These results help identify sex and APOE ε4 genotype-specific transcriptomic signatures of AD and underscore the importance of considering sex in the development of biomarkers and therapeutic strategies for AD.

## Introduction

Alzheimer’s disease (AD) is a progressive neurodegenerative disorder and the most common cause of dementia (Bekris et al., 2010; Alzheimer’s Association, 2018). It is pathologically characterized by the deposition of extracellular amyloid β (Aβ) and intracellular tau, otherwise referred to as plaques and neurofibrillary tangles, respectively (Hardy and Selkoe, 2002; Holtzman et al., 2011; Karch and Goate, 2015). AD is also marked by neuronal loss, impaired neurotransmitter signaling, neuroinflammation, and dysregulation of neuronal metabolism and immune response in the central nervous system (Torrão et al., 2012; Riedel et al., 2016; Medeiros and Silva, 2019). AD prevalence increases dramatically with age, where the majority of cases are in individuals above the age of 65 (Hebert et al., 2013; Alzheimer’s Association, 2018). Although AD was identified more than a century ago (Goedert and Spillantini, 2006), its cause and pathophysiology are not fully understood, and there are no available treatments that aid in halting or reversing the disease (Cummings et al., 2014). Accordingly, it is of high priority to tackle AD, as it is projected to triple in incidence by 2050 as a consequence of population aging (Riedel et al., 2016; Medeiros and Silva, 2019; United Nations, 2019) and, to date, has no disease-modifying therapies.

While the exact cause and pathophysiology remain unknown, a number of mutations and genetic risk factors have been identified as associated with AD. Apolipoprotein E (APOE) is the most common genetic risk factor for late onset AD (Mahley et al., 2006; Peskind et al., 2009; Holtzman et al., 2012; Huang and Mucke, 2012; Riedel et al., 2016; Liu et al., 2019; Paranjpe et al., 2019a). ApoE is a lipid binding protein, that plays a central role in lipid transport and metabolism. It is highly expressed in the brain, and is important for maintaining neuronal membranes during inflammation and damage. In humans, APOE has three isoforms, APOE ε2, APOE ε3, and APOE ε4, which are encoded by the three alleles, ε2, ε3, and ε4, of the APOE gene, respectively. The ε2 isoform has been shown to be protective against AD, while the ε4 isoform (APOE ε4) is associated with increasing the risk and lowering the age of onset for developing late onset AD in a gene dose-dependent manner (Saunders et al., 1993; Corder et al., 1994). Specifically, one copy of the ε4 isoform confers a 3 to 4-fold increased risk and 7 year decrease in age of onset, while two copies confers a 12 to 15-fold increased risk of AD, and a 14 year decrease in age of onset (Corder et al., 1993; Riedel et al., 2016).

Sex is another major risk factor in AD. Female sex is associated with increased AD incidence, exacerbated pathophysiology and increased rate of cognitive decline related to the disease progression (Andersen et al., 1999; Barnes et al., 2005; Carter et al., 2012; Irvine et al., 2012; Riedel et al., 2016; Nazarian et al., 2019; Zhao et al., 2020). It has been conjectured that the higher prevalence in females is a result of longer life span (Carter et al., 2012; Riedel et al., 2016). Alternatively, studies have alluded to sex-specific hormonal and metabolic changes that interplay with the onset and progression of AD dementia (Altmann et al., 2014; Liu et al., 2019; Medeiros and Silva, 2019). Sex also interacts with APOE isoform status, where females with the APOE ε4 isoform are at increased risk compared to males (Farrer, 1997; Bretsky et al., 1999; Ungar et al., 2014). Despite the clear therapeutic potential to better understand these pathophysiological patterns, there is still little understanding of the mechanisms underlying sex-specific differences in AD.

**FIGURE 1:**
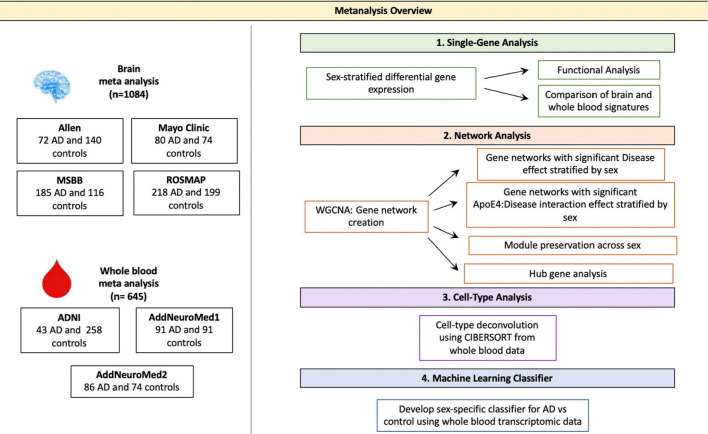
Meta-analysis overview. Diagram depicting the study overview including all datasets used and analyses performed. Data sets were obtained via searching GEO or PubMed for the keyword Alzheimer’s Disease. Samples with neurological conditions other Alzheimer’s, including Parkinson’s Disease and Huntington’s Disease, and single cell preparations were excluded from analysis. Datasets were merged using the ComBat package in R. WGCNA was used for network analyses. CIBERSORT was used for cell type deconvolution. The linear SVM was trained to classify AD and control patients using the transcriptomic signature obtained via meta-analysis of blood studies. The performance of a molecular model consisting of gene expression, age, sex and APOE ε4 status was compared to that clinical model with age, sex and APOE ε4 status as features.

With the rising prevalence of AD, it is critical to facilitate the development of robust means to detect AD early and discover therapeutic interventions (Cummings et al., 2019a,b; Paranjpe et al., 2019b; Huang et al., 2020). Technological innovations and the increasing availability of large transcriptomic datasets present worthwhile avenues to study and characterize the molecular underpinnings of AD stratified by sex. Here, we analyze publicly available gene expression datasets from over 1,500 brain and blood samples to characterize this highly complex disease. To derive sex-specific transcriptomic molecular signatures, we perform a meta-analysis, differential gene expression, weighted gene co-expression network analysis, pathway enrichment, and cell-type deconvolution in a large cohort of brain and blood samples from AD patients and healthy controls (Figure 1). We further characterize these signatures and apply machine learning to build a predictive model based on biomarkers identified in the blood of AD patients. Our findings reveal sex-associated gene expression patterns in AD, which provide clinical implications for identifying more accurate, and less invasive biomarkers, as well as efficacious therapeutics tailored to better fit the complex molecular profiles in AD.

## Materials and Methods

### Study Cohorts

Publicly available RNA-sequencing (RNA-Seq) and microarray datasets from the Gene Expression Omnibus (GEO) and from consortium studies indexed on PubMed were searched for the key word “Alzheimer’s.” Relevant studies were filtered for a high quality study design and were required to contain clinical metadata including age, sex, APOE ε4 carrier status, and education. To minimize technical variability, brain samples were restricted to RNA-sequencing studies while blood analyses were restricted to microarray studies. Samples were curated to include bulk gene expression from subjects with Alzheimer’s or elderly healthy individuals with no history of neurodegenerative disease. Individuals with non-Alzheimer’s neurodegenerative diseases including Huntington’s and Parkinson’s were excluded. Brain samples were restricted to the hippocampus, parietal cortex, temporal cortex and prefrontal cortex. Clinical metadata including age, sex, APOE ε4 carrier status, education were recorded for the samples and used as covariates or stratification variables in subsequent analyses.

### Gene Expression Meta-Analysis

Meta-analysis was conducted separately for brain and blood studies according to standard quality control, normalization, and batch correction procedures. All data processing was conducted using R (v3.6.1).

#### Brain Studies

Raw RNA-sequencing data were processed for the Mount Sinai Brain Bank (MSBB; Wang et al., 2018), Mayo Clinic RNAseq (Allen et al., 2016), and Religious Orders Study and Memory and Aging Project (ROSMAP; Bennett et al., 2012) as previously described in the AMP-AD consortium project. Briefly, read alignment and counting was performed using STAR (Dobin et al., 2013). Alignment quality metrics were generated using Picard (2019). For the Allen dataset, expected counts produced using RSEM were downloaded from the Allen Brain Atlas: Aging Dementia and TBI Study website (Aging, Dementia and TBI Study, 2019). Counts-per-million (CPM) were calculated for all studies. Genes with less than 1 CPM in at least 50% of samples across tissue diagnosis group were removed. Genes with missing gene length or GC content percentage metrics were removed. Library normalization was performed using conditional quantile normalization.

Following read alignment and normalization, studies were merged using common genes between the four studies. Mean value imputation was performed for missing gene expression values. Quantile normalization was performed across studies. The ComBat function from the *sva* package (Leek et al., 2012) was used to perform cross-study normalization, retaining variation in APOE ε4 carrier status, sex, and diagnosis. Principal component analysis (PCA) plots were generated to evaluate the success of batch correction and to detect outliers.

#### Blood Studies

Study data were downloaded from GEO for the AddNeuroMed datasets (Hodes and Buckholtz, 2016) or the Alzheimer’s Disease Neuroimaging Initiative Consortium (ADNI) (Petersen et al., 2010) for the ADNI dataset and processed. Raw data were not available for the ADNI dataset and therefore normalized expression data were used for all studies. Outlier removal was performed on individual studies by removing probes whose mean expression was outside 1.5 times the interquartile range. Probe IDs were mapped to gene symbols. Expression value of probes mapping to the same gene were reported as the median of all probes mapping to that gene (Zhang, 2016). Quantile normalization was performed across studies. Similar to the brain data analysis, the ComBat function from the *sva* package was used to perform cross-study normalization, retaining variation in APOE ε4 carrier status, sex and diagnosis. Principal component analysis (PCA) plots were generated to evaluate successful batch correction.

Data used in the preparation of this article were partly obtained from the Alzheimer’s Disease Neuroimaging Initiative (ADNI) database.^1^ The ADNI was launched in 2003 as a public-private partnership, led by Principal Investigator Michael W. Weiner, MD. The primary goal of ADNI has been to test whether serial magnetic resonance imaging (MRI), positron emission tomography (PET), other biological markers, and clinical and neuropsychological assessment can be combined to measure the progression of mild cognitive impairment (MCI) and early Alzheimer’s disease (AD).

### Differential Gene Expression Analysis

All differential gene expression analyses were performed separately for brain and blood samples. The Limma package (Ritchie et al., 2015) was used to determine differentially expressed genes between cases and controls all together and stratified by sex. In each model, age and APOE ε4 carrier status were included as covariates to minimize confounding. An additional covariate of education was used in the blood analyses. Education was not available for all brain samples and therefore was not included as a covariate. A cutoff false discovery rate (FDR) of 0.05 and fold change (FC) of greater than or equal to 1.2 was used for brain analyses. Fold changes were calculated using the individual study data before merging and weighted by sample size. For blood analyses, a FC cutoff was not used to maximize gene discovery, given that we expect signals to be considerably lower in the periphery than we do in disease tissue. Significant overlap between up- and down-regulated genes between males and females was assessed using a hypergeometric test. Functional enrichment analysis of gene lists was carried out by overrepresentation analysis using the KEGG (Kanehisa and Goto, 2000) database of biological pathways.

### Network Analysis

#### Weighted Gene Co-expression Network Analysis

In order to detect gene network level differences, network analysis was performed using Weighted Gene Co-Expression Network Analysis (WGCNA; Langfelder and Horvath, 2008). All analyses were performed separately for brain and blood samples. In signed WGCNA, a module was defined as a set of genes whose expression is highly correlated in the same direction. In brief, pairwise, signed similarity matrices were computed separately for male and female gene expression profiles. Pairwise similarity between two gene expression profiles, *x*_*i*_ and *x*_*j*_ was defined as:


$$s_{i,j}^{signed}=0.5+0.5cor\left(x_{i},x_{j}\right)$$

An adjacency matrix was computed by raising the pairwise similarity matrix to a power, β, defined as the minimum value required for the network to achieve a scale-free topology. The adjacency matrix was transformed into a Topological Overlap Matrix as previously described (Langfelder and Horvath, 2008). To identify clusters of interconnected genes, termed modules, hierarchical clustering was performed on Topological Overlap Matrix and modules were selected using the Dynamic Branch Cutting approach, as previously described (Langfelder and Horvath, 2008).

Module Z-summary scores were computed to assess module preservation between male and female networks, as described previously (Langfelder et al., 2011). A Z-summary score greater than ten was considered to be strong evidence of preservation between the two networks. A score between two and ten was considered to represent weak to moderate evidence of preservation, as previously described (Langfelder et al., 2011).

Association between module gene expression and case/control status was assessed by relating the module eigengenes, defined as the first principal component of the genes in a given module, to case/control status using linear regression. Age, APOE ε4 carrier status, and education (for blood samples) were used as covariates to minimize confounding. An additional analysis identifying apoE-by-disease interaction effects was performed by adding the interaction term: APOE ε4 carrier status:case/control status to the previous model. Significant modules were characterized by performing functional gene enrichment using the KEGG database of biological pathways (Farrer, 1997).

#### Hub Gene Analysis

To identify central regulators of gene expression, we identified hub genes within significant modules, as described previously (Langfelder and Horvath, 2008). Hub genes were defined as genes with gene significance (the correlation between the gene expression and case/control status) greater than 0.2 and module membership (the correlation between gene expression and module eigengene) greater than 0.8, as previously described (Langfelder and Horvath, 2008). We also restricted hub genes to those that were differentially expressed in AD vs. control. Network visualization using the STRING v11 (Szklarczyk et al., 2019) database was used to assess evidence for protein-protein interactions between hub genes.

### Cell-Type Deconvolution

CIBERSORT (Newman et al., 2015) was applied to the transcriptomic signatures generated in the blood meta-analysis to deconvolve gene expression data into cell type composition and identify sex-specific dysregulation of immune cell types between cases and controls. CIBERSORT applies a linear support vector regression method to solve the problem: *m* = f x B where m is an input mixture of gene expression data for a given sample, f is a vector consisting of fractions of each cell type in the mixture and B is a matrix of reference gene expression profiles. A gene expression profile of 22 reference cell populations was built using differential gene expression of purified or enriched cell populations from the authors of CIBERSORT.

CIBERSORT was used to deconvolve gene expression data from pooled male and female data, male only samples, and female only samples. In each condition, differences in cell type proportions between cases and controls were compared using a linear regression model adjusting for age, sex (in the pooled male female analysis), and APOE ε4 carrier status. An additional analysis identifying APOE ε4-by-disease interaction effects was performed by adding the interaction term: APOE ε4 carrier status:case/control status to the previous model. A cutoff FDR of 0.05 was deemed significant.

### Classification of Healthy and Alzheimer’s Disease Patients

A linear support vector machine (SVM) model with *l*_1_ regularization to enforce feature sparsity was used to classify Alzheimer’s patients and healthy controls based on blood gene expression data. To assess the relative value of stratifying by sex in increasing model performance, we compared the performance of three models built using pooled male and female samples, male samples only, and female samples only. We compared the performance of a ‘clinical model’ with age, sex (for male and female pooled samples), and APOE ε4 carrier status information to a ‘clinical + molecular model’ which included age, sex (for male and female pooled samples), APOE ε4 carrier status, and transcriptomic data from the blood meta-analysis. For models with transcriptomic data, we included gene expression data from the corresponding sex. For example, the clinical+molecular using female samples only included gene expression data from females.

For each model, data were split into 75% training/validation and 25% test sets using a class balancing procedure to maintain a constant case/control ratio across training/validation and test sets. A random search over the space 10^–4^ to 10^4^ with five-fold cross validation was used to optimize the C hyper-parameter, or the degree of regularization penalty applied for misclassified points. Receiver operating characteristic (ROC) curves were generated from the test set. Model performance was assessed using the area under the ROC curves. Feature importance was determined using the absolute value of the model coefficients corresponding to the vector coordinates orthogonal to the model hyperplane.

### Down Sampling Sensitivity Analysis

Given the imbalance in the proportion of AD cases in the female samples compared to male samples, we down sampled our brain and blood datasets to assess whether our results were primarily driven by differences in statistical power between males and females. Specifically, we performed 100 iterations of down sampling. In each iteration, we down sampled the female samples in our dataset such that the total number of AD cases and controls was the same in the male and female groups. For example, the number of cases and controls in the original dataset and down sampled dataset for the brain data are presented below:

  
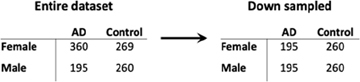


In each iteration, we calculated the number of differentially expressed genes in males and females. We then randomly selected one iteration to repeat all the other analyses, including functional enrichment analysis, network analyses, SVM-based classification and cell type deconvolution, that were performed on the dataset without down sampling to assess the contribution of statistical power to our findings.

## Results

### Study Cohort Characteristics

We obtained four publicly available RNA-seq data sets (Allen Brain Institute Aging Dementia and TBI study, Mayo Clinic RNA-seq, MSBB, and ROSMAP) from the brain (temporal cortex, parietal cortex, prefrontal cortex, and hippocampus) and three microarray datasets from whole blood (AddNeuroMed cohort 1, AddNeuroMed cohort 2 and ADNI). After outlier removal, we included a total of 1,084 brain samples (58% female; 26% APOE ε4 carriers) and 645 blood samples (58% female; 38% APOE ε4 carriers) in our analysis. Table 1 shows a summary of sample annotations including number of cases and controls, APOE ε4 carrier status, and number of males and females for brain datasets and blood datasets.

**TABLE 1:** Meta-analysis study characteristics.

				AD		CN	
Study	Accession	Total participants	AD, no. (%)	Female/Male (% Female)	APOE ε4 Carrier Yes/No (% Yes)	Female/Male (% Female)	APOE ε4 Carrier Yes/No (% Yes)
**Brain Transcriptomic Studies**							
Allen	https://aging.brain-map.org/	212	72 (34)	29/43 (40)	22/50 (31)	54/86 (39)	19/121 (14)
Mayo Clinic RNA-Seq	syn5550404	154	80 (52)	49/31 (61)	42/38 (53)	36/38 (49)	9/65 (12)
MSBB	syn3159438	301	185 (62)	131/54 (71)	63/122 (34)	57/59 (49)	16/100 (13)
ROSMAP	syn3219045	417	218 (52)	151/67 (70)	83/135 (38)	122/77 (61)	33/166 (17)
**Sum**		**1084**	**555 (52)**	**360/195 (65)**	**210/345 (38)**	**269/260 (51)**	**77/452 (15)**
**Whole Blood Transcriptomic Studies**							
ADNI	http://adni.loni.usc.edu/	301	43 (14)	17/26 (40)	32/11 (74)	135/125 (52)	71/189 (27)
AddNeuroMed1	GSE63060	182	91 (50)	65/26 (71)	52/39 (57)	55/36 (60)	30/61 (33)
AddNeuroMed2	GSE63061	160	86 (43)	59/27 (69)	47/39 (55)	45/29 (61)	15/59 (20)
**Sum**		**645**	**220 (34)**	**141/79 (64)**	**131/89 (60)**	**235/190 (55)**	**116/309 (27)**

In the brain datasets, compared to controls, AD patients were significantly older (mean ± SD for AD: 86.5 ± 6.0 years and controls: 84.8 ± 7.4 years; two sample *t*-test, *P* < 0.001), more likely to be APOE ε4 carriers (AD: 38% carriers vs controls: 15% carriers; Chi-squared test, *P* < 0.001), and more likely to be females (AD: 65% female vs controls: 51% female; Chi-squared test, *P* < 0.001). The proportion of male and female samples from each brain region differed significantly across brain regions (Chi-squared test, *P* < 0.01), as summarized in Supplementary Table S1.

In the blood datasets, compared to controls, AD patients were significantly older (mean ± SD for AD: 77.0 ± 7.1 years and controls: 74.7 ± 5.7 years; two sample *t*-test, *P* < 0.001), more likely to be APOE ε4 carriers (AD: 60% carriers vs controls: 27% carriers; Chi-squared test, *P* < 0.001), more likely to be females (AD: 64% female vs controls: 55% female; Chi-squared test, *P* < 0.001), and had fewer years of education (mean ± SD for AD: 9.4 ± 4.8 years and controls: 13.9 ± 4.7 years; two sample *t*-test, *P* < 0.001).

Studies were merged and batch corrected using ComBat resulting in 13,345 common genes across 1,084 samples for brain studies and 3,371 common genes across 645 samples for blood studies. Supplementary Figures S1, S2 show PCA plots before and after batch correction, demonstrating successful data merging and batch effect removal. Additionally, Supplementary Figure S3 demonstrates that the gene expression distribution does not differ across female and male samples in either the brain dataset (Supplementary Figure S3A; KS test, *p* > 0.05) or blood dataset (Supplementary Figure S3C; KS test, *p* > 0.05). Coefficients of variation were similar across male and female samples in both the brain (Supplementary Figure S3B; *t*-test *p* > 0.05) and blood (Supplementary Figure S3D; *t*-test *p* > 0.05) datasets.

### Differential Gene Expression in the Brain Identifies a Distinct Sex-Specific Signature of AD

**FIGURE 2:**
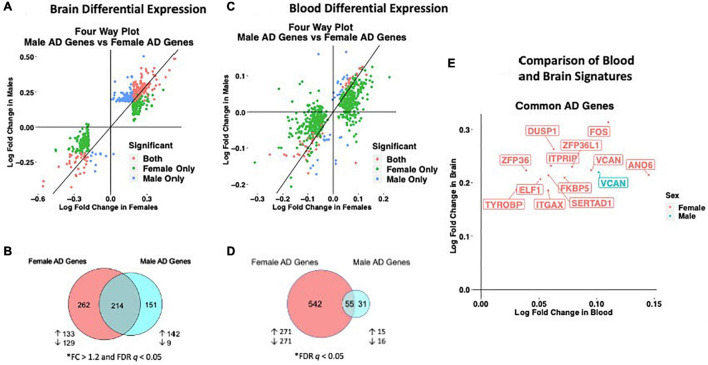
Cross-tissue sex specific differential gene expression. **(A)** Four-way plot with fold change in males vs fold change in females depicting differentially expressed genes in the brain. Differential expression was defined using a fold change > 1.2 and FDR *P* < 0.05. Covariates of age and sex were included in statistical analyses. **(B)** In the brain, a total of 631 genes were uniquely dysregulated in females with AD while 166 genes were uniquely dysregulated in males with AD. Common to both males and females in the brain were 343 genes. **(C)** Four-way plot with fold change in males vs fold change in females depicting differentially expressed genes in the blood. Differential expression was defined using a fold change > 1.2. Covariates of age, sex, and education were included in statistical analyses. **(D)** A total of 542 genes were uniquely dysregulated in females with AD while 31 genes were uniquely dysregulated in males with AD in blood. Common to both males and females in the brain were 55 genes. **(E)** Fold change plot depicting genes that are dysregulated in both blood and brain tissues. Genes are colored by sex indicating if the gene is dysregulated in male samples (1 gene; red) or female samples (31 genes; blue).

We observed distinct AD-associated transcriptomic signatures in the brain in males and females. A total of 476 genes were differentially expressed in females, including 306 upregulated genes and 170 downregulated genes (FC > 1.2, *q* < 0.05; Figures 2A,B; Supplementary Table S2). In males, 365 genes were differentially expressed, including 318 upregulated genes and 47 downregulated genes (FC > 1.2, *q* < 0.05; Figures 2A,B; Supplementary Table S2). Altogether, 262 genes were uniquely dysregulated in females, including 133 upregulated genes and 129 downregulated genes. In males, 151 genes were uniquely dysregulated, including 142 upregulated genes and 9 downregulated genes. There was a significant overlap of dysregulated genes across males and females (*P* < 0.05; hypergeometric test).

Next, we characterized the transcriptomic signatures observed in the brains of male and female AD patients. In females, among upregulated AD genes, we found 46 enriched pathways, some of them suggesting dysregulation in components of the innate and adaptive immune system (Table 2; Supplementary Table S3). Several upregulated HLA system genes including HPA-DRA and HLA-DPA1 contributed to enrichment of a number of pathways relating to response to infection (Table 2). Components of the complement system including C3AR1, C4B, and C4A were also uniquely dysregulated in females (Table 2; Supplementary Table S3). We also observed an enrichment of genes in the MAPK signaling pathway including MRAS and MK2. Downregulated AD genes in females were enriched for a number of neurological signaling pathways including GABAeric signaling, neuroactive ligand-receptor activation, and cAMP signaling (Table 2; Supplementary Table S4).

**TABLE 2:** Enriched pathways in the brain.

Term	Adjusted P	Genes
**Female Upregulated Genes (*n* = 583)**		
Malaria	<0.001	TGFB2;TGFB1;GYPC;HGF;ITGB2;PECAM1;CCL2;TLR4;ICAM1
Hippo signaling pathway	<0.001	YAP1;CRB2;WWTR1;TGFB2;TGFB1;FZD7;SERPINE1;ITGB2;BMP6;GLI2;TGFBR2;PARD3;CCN2;AJU BA;TEAD2
PI3K-Akt signaling pathway	<0.001	NGFR;CDKN1A;ANGPT2;CSF1;ITGB5;ITGB4;LAMB2;HGF;IGF2;GNG12;OSMR;PGF;PIK3R5;COL1A2; ITGA10;COL6A2;DDIT4;CDK2;SPP1;ITGA5;TLR4
Proteoglycans in cancer	<0.001	CDKN1A;TGFB2;TGFB1;HPSE2;ITGB5;FZD7;HGF;IGF2;DCN;MRAS;SMO;ITGA5;EZR;TLR4;CD44
Human T-cell leukemia virus 1 infection	<0.001	CDKN1A;TGFB2;TGFB1;ITGB2;NFATC2;FOS;ICAM1;NFATC4;TGFBR2;NFKBIA;ZFP36;CDK2;HLA-DRA; MSX1;HLA-DPA1
Rheumatoid arthritis	<0.001	TGFB2;TGFB1;CSF1;ITGB2;CCL2;HLA-DRA;FOS;TLR4;ICAM1;HLA-DPA1
ECM-receptor interaction	<0.001	COL1A2;ITGB5;ITGB4;LAMB2;COL6A2;ITGA10;SPP1;ITGA5;CD44
Osteoclast differentiation	<0.001	NFKBIA;SOCS3;TGFB2;TYROBP;TGFB1;CSF1;NFATC2;TNFRSF11B;TREM2;FOS;TGFBR2
TGF-beta signaling pathway	<0.001	TGIF1;TGFB2;TGIF2;TGFB1;ID4;ID3;DCN;BMP6;TGFBR2
Staphylococcus aureus infection	<0.001	C4B;C4A;ITGB2;CFI;C3AR1;HLA-DRA;ICAM1;HLA-DPA1
36 more..		
**Female Downregulated Genes (*n* = 398)**		
Neuroactive ligand-receptor interaction	<0.001	GABRA1;CHRM4;SSTR1;TACR1;HTR5A;RXFP1;GABRG2;MCHR2;ADCYAP1;MAS1;GLRA3;CCKBR; SST;GALR1;TAC3;TAC1;VIP
GABAergic synapse	0.002	PRKCG;GABRA1;GNG3;SLC32A1;GAD1;GAD2;GABRG2
cAMP signaling pathway	0.009	ADCYAP1;PAK1;BDNF;SST;CAMK4;CALM3;SSTR1;VIP;CNGB1
African trypanosomiasis	0.02	PRKCG;HBB;HBA2;HBA1
**Male Upregulated Genes (*n* = 415)**		
Focal adhesion	<0.001	VAV3;PDGFRB;FLT1;ITGB5;LAMB2;HGF;CAV1;FN1;ELK1;PGF;COL1A2;ITGA10;COL6A2;SPP1;IT GB8;ITGA5;TLN1
PI3K-Akt signaling pathway	<0.001	PDGFRB;NGFR;CDKN1A;FLT1;ANGPT2;CSF1;ITGB5;LAMB2;HGF;FN1;IGF2;PGF;PIK3R5;COL1A2; ITGA10;COL6A2;DDIT4;SPP1;ITGB8;ITGA5;TLR4;EPHA2
Proteoglycans in cancer	<0.001	CDKN1A;TGFB2;ITGB5;HGF;CAV1;MMP2;IGF2;FN1;IQGAP1;ELK1;DCN;SMO;ITGA5;EZR;TLR4;CD44
ECM-receptor interaction	<0.001	COL1A2;ITGB5;LAMB2;COL6A2;ITGA10;SPP1;FN1;ITGB8;ITGA5;CD44
MAPK signaling pathway	0.001	PDGFRB;NGFR;TGFB2;FLT1;ANGPT2;CSF1;DUSP1;HGF;IGF2;HSPB1;ELK1;PGF;TGFBR2;GNA12; EPHA2;HSPA1A
Hippo signaling pathway	0.01	YAP1;CRB2;WWTR1;TGFB2;LATS2;CCN2;BMP6;TEAD2;GLI2;TGFBR2
Pathways in cancer	0.02	PDGFRB;NOTCH2;CDKN1A;CDKN2B;TGFB2;LAMB2;HGF;MMP2;FN1;IGF2;LRP5;CXCR4;ELK1; PGF;GLI2;TGFBR2;NFKBIA;CASP7;SMO;GNA12
Ras signaling pathway	0.02	PDGFRB;NGFR;FLT1;ANGPT2;CSF1;HGF;IGF2;FOXO4;ELK1;PGF;EPHA2;PLA1A
TGF-beta signaling pathway	0.02	TGFB2;CDKN2B;ID3;DCN;BMP6;RGMA;TGFBR2
Regulation of actin cytoskeleton	0.02	VAV3;PDGFRB;ITGB5;ITGA10;GNA12;FN1;CXCR4;ITGB8;IQGAP1;ITGA5;EZR
4 more..		
**Male Downregulated Genes (*n* = 98)**		
Malaria	0.02	HBB;HBA2;HBA1
Neuroactive ligand-receptor interaction	0.02	MAS1;ADCYAP1;SST;TAC3;TAC1;VIP
Taurine and hypotaurine metabolism	0.02	GAD1;GAD2
African trypanosomiasis	0.03	HBB;HBA2;HBA1

Strikingly, we observed an enrichment of fewer immune-related pathways in males with AD. Among upregulated genes in male AD patients, we found 12 enriched pathways, including amoebiasis and cytokine-cytokine receptor interaction, suggestive of adaptive and innate immune activation (Table 2; Supplementary Table S5). Similar to females, we also observed an enrichment of the MAPK signaling pathway, including MAP4K4 and MK2, in males. Among downregulated genes in male AD patients, we observed an enrichment of neuropeptide signaling and glutamate signaling related pathways (Table 2; Supplementary Table S6). For a full list of enriched pathways, refer to Supplementary Tables S3–S6.

Lastly, we performed a non-stratified analysis comparing gene expression between AD and control samples irrespective of sex. Statistical models were adjusted for sex, APOE ε4 carrier status, and age. A total of 662 genes were upregulated and 430 genes were downregulated in patients with AD compared to controls (Table 2; Supplementary Figure S4; Supplementary Table S2. Upregulated genes were enriched for several pathways previously implicated in AD including PI3K-Akt signaling and MAPK signaling as well as a number of immune related pathways including Staphylococcus aureus infection, human papillomavirus infection, and malaria (Supplementary Table S7). Several components of the complement system, including C4B, C4A, C1R, C3AR1, and C5AR1 also contributed to this enrichment (Supplementary Table S8). In our analysis of downregulated genes, we found several pathways related to neuroreceptor signaling and GABAergic transmission were enriched including the genes GABRA1, GNG3, GNG2, SLC32A1, GABRD, and GABRG2 (Supplementary Table S8).

### Network Analysis in the Brain Identifies a Stronger Disease Signature in Females

**FIGURE 3:**
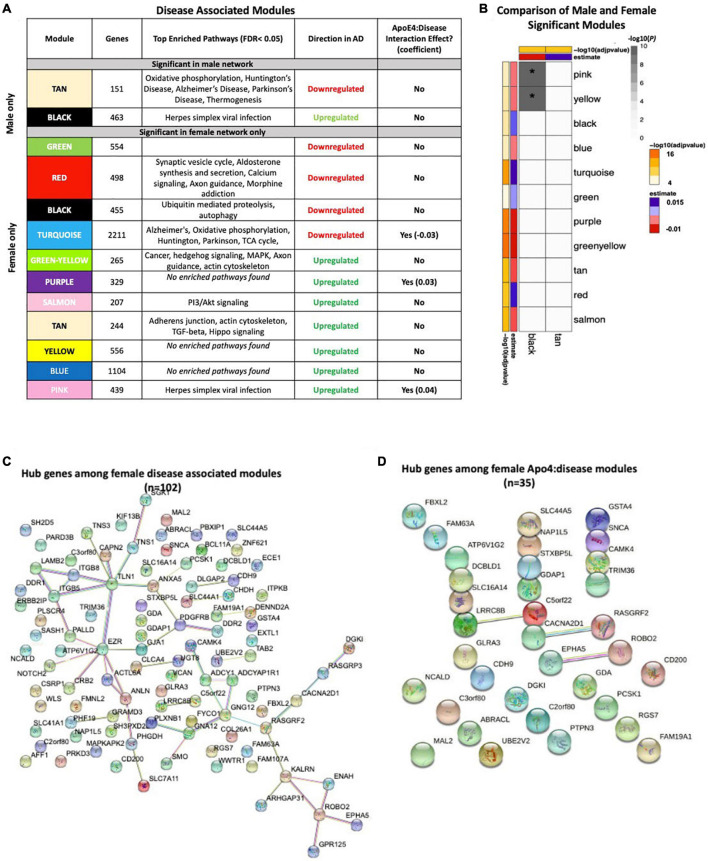
Network analysis in the brain. WGCNA was used to construct gene network separately for males and females in the brain. Networks were randomly assigned colors. **(A)** A description of the disease-associated gene networks (termed modules) produced using WGCNA. Significant disease-associated modules were identified by associating module eigengene to case/control status adjusting for age and APOE ε4 status (*P* < 0.05). KEGG enrichment analysis of significant was conducted using an adjusted *P-*value threshold of 0.05. The direction in AD is computed using the case/control coefficient of the model associating module eigengene to case/control status. Modules with significant APOE ε4 :disease interaction effect were identified by adding the interaction term APOE ε4 :disease to the previous model (*P* < 0.05). **(B)** Heatmap depicting the degree of module overlap assessed using a hypergeometric test between male and female disease-associated modules. The black module in males had significant overlap (*P* < 0.05) with the pink and yellow modules, indicated by * in the heatmap. Estimate and −log10(adjpvalue) refers to the case/control coefficient and *p*-value in the model: module eigengene ∼ age + APOE ε4 + case/control status. **(C)** Hub genes from female disease-associated modules. Hub genes were defined as genes with gene significance (the correlation between the gene expression and case/control status) greater than 0.2 and module membership (the correlation between gene expression and module eigengene) greater than 0.8. Hub genes were restricted to those that were differentially expressed in AD vs control. Protein-protein interactions between hub gene visualization was performed using the STRING v11 database. Edge color represents the type of interaction evidence for protein-protein interaction (cyan: known interaction from curated databases; turquoise: experimentally determined; green: gene-neighborhood predicted interaction; red: gene-fusions predicted interaction; blue: gene co-occurrence predicted interaction; green-yellow: text mining; black: co-expression; light purple: protein homology. **(D)** Hub genes among modules with significant APOE ε4: disease interaction effect. Protein-protein interaction between hub genes was visualized using STRING v11 with edge colors representing the same as in panel **(C)**.

To assess transcriptomic changes on a gene network level, we utilized WGCNA. Gene networks were derived separately for male and female samples and compared using network preservation methods, as previously described (Langfelder et al., 2011). We identified two AD-associated modules in males and 11 AD-associated modules in females (Figure 3A) that met the significance threshold (FDR < 0.05) and were either positively or negatively correlated with case/control status. Among the male modules, a 463-gene module (termed black) was upregulated in AD, and a 151-gene module (termed tan) was downregulated in AD. The black module in males had significant overlap with two modules in females (termed yellow and pink) (*P* < 0.001; hypergeometric test) as indicated by asterisks in Figure 3B. The black module also had strong preservation in the female network (*Z*-summary score > 10). Similarly, the tan module had strong preservation in the female-network (*Z*-summary score > 10). Among the female-specific disease associated modules, four modules (termed green, red, black and turquoise) were downregulated in AD, while seven were upregulated (Figure 3A).

Enrichment analysis of disease-associated modules using the 2019 KEGG Human pathway database revealed pathways relevant to AD that were consistent with those identified in the single gene analysis (Figure 3A). For example, in both males and females, an upregulated module was enriched for Akt signaling related pathways and downregulated modules were enriched for oxidative phosphorylation and thermogenesis related pathways, consistent with single gene level analyses.

Notably, several additional pathways not seen through single gene analysis were observed in the network analyses. An upregulated module in both males and females was highly enriched for zinc finger nuclease genes related to Herpes simplex viral infection, consistent with recent work demonstrating Herpes virus infection in AD brains (Itzhaki, 2018).

Consistent with the single gene analysis, we observed greater number of disease associated modules in females with AD than in males. For example, an upregulated female module was enriched for cell structural processes related to adherens junctions, actin cytoskeleton and axonal guidance. An additional downregulated female module was enriched for neurological signaling pathways including synaptic vesicle exocytosis, aldosterone synthesis and secretion and morphine addiction. Interestingly, an additional female downregulated module was enriched for autophagy and proteolysis pathways, consistent with molecular studies demonstrating decreased autophagy in AD, particularly in females (Congdon, 2018; Figure 3A).

We also conducted an analysis identifying modules with APOE ε4:disease interactive effect to understand differential penetrance of the apoE ε4 allele in males and females. In the male gene network, we were unable to identify modules with significant APOE ε4:disease interactive effect. Interestingly, in the female network, we identified one module that was downregulated (2211 genes) in AD, and two modules (329 genes and 439 genes) that were upregulated in AD and exhibited a significant APOE ε4:disease interactive effect (Figure 3A). The two upregulated modules (termed pink and purple) were significantly enriched for several zinc finger nuclease genes related to Herpes simplex viral infection. The downregulated module was enriched for metabolic pathways including oxidative phosphorylation and the TCA cycle. Together these results suggest a female-specific network dysregulation involving zinc finger nucleases and metabolic alteration supporting differential APOE ε4 penetrance in males and females.

There were 102 hub genes among disease associated modules in the female network identified as module membership greater than 0.8, gene significance greater than 0.2, and differentially expressed between AD and controls (Figure 3C; Supplementary Table S9). In contrast, zero hub genes were identified in the male gene network. Protein-protein interaction maps generated by STRING v11 suggest several Ca^+2^- and G protein-dependent interconnected genes including ITPKB, PDGFRB, GNG12, and GNA12 among the female disease associated modules (Figure 3C). Among modules with APOE ε4:disease interactive effect in females, 35 hub genes were identified, including ITPKB as a highly connected regulator (Figure 3D). For a full list of genes in each module, including hub genes, please refer to Supplementary Table S9).

### Differential Gene Expression in Whole Blood Identifies Stronger Disease Signatures in Females With AD in Comparison to Males

Similar to the brain, we observed distinct AD-associated transcriptomic signatures between males and females with AD in whole blood. We observed a total of 597 differentially expressed genes in females with AD, including 294 upregulated genes and 303 downregulated genes (*q* < 0.05; Figures 2C,D; Supplementary Table S10). In males, 86 genes were differentially expressed in AD, including 36 upregulated genes and 50 downregulated genes (*q* < 0.05; Figures 2C,D; Supplementary Table S10). Altogether, 542 genes were uniquely dysregulated in females, including 271 upregulated genes and 271 downregulated genes. In males, 31 genes were uniquely dysregulated, including 15 upregulated genes and 16 downregulated genes. There was a significant overlap of dysregulated genes across males and females with AD (*P* < 0.05; hypergeometric test).

Next, we characterized the transcriptomic signatures observed in the blood of male and female AD patients. Among upregulated genes in female AD patients, we found 14 enriched pathways, many of them relating to components of the innate and adaptive immune system (Table 3; Supplementary Table S11). Several cytokine response elements including STAT5B, STAT6, and IL10RB contributed to enrichment of a number of pathways relating to response to infection (Table 3). Similar to the brain, components of actin cytoskeleton regulation were also dysregulated in females (Table 3; Supplementary Table S11). Downregulated genes in female AD patients were enriched for a number of metabolism related processes including oxidative phosphorylation and thermogenesis, consistent with the single-gene and network analysis in the brain (Supplementary Table S12).

**TABLE 3:** Enriched pathways in blood.

Term	Adjusted P	Genes
**Female Upregulated Genes (*n* = 294)**		
Tuberculosis	<0.001	ATP6V0B;CEBPB;ITGAM;IL10RB;IFNGR2;TCIRG1;CTSS;CREB1;IRAK1;LAMP2;ITGAX;RAF1; CAMK2G
Necroptosis	0.004	PYCARD;STAT5B;MLKL;H2AFJ;IFNGR2;STAT6;TYK2;CFLAR;CAMK2G;HIST1H2AC;HIST2H2AC
Fc gamma R-mediated phagocytosis	0.006	HCK;PTPRC;ARPC1A;PRKCD;RAC2;ASAP1;ARPC5;RAF1
Pathogenic Escherichia coli infection	0.01	ARPC1A;NCK2;ARHGEF2;ARPC5;TLR5;TUBA4A
TNF signaling pathway	0.01	CEBPB;RPS6KA5;CREB1;MLKL;MAP3K8;FOS;CFLAR;CREB5
Regulation of actin cytoskeleton	0.02	FGD3;ITGAM;SPATA13;ARPC1A;RAC2;ITGAX;IQGAP1;ARPC5;RAF1;SSH2;PAK2
Lysosome	0.02	GNPTG;CD63;ATP6V0B;LAMP2;IDS;TCIRG1;GNS;CTSS
Phagosome	0.02	ATP6V0B;ITGAM;LAMP2;CANX;TAP1;TCIRG1;TUBA4A;CTSS;ATP6V1F
JAK-STAT signaling pathway	0.02	STAT5B;CCND3;CSF3R;IL10RB;IFNGR2;STAT6;TYK2;RAF1;MCL1
Estrogen signaling pathway	0.03	CREB1;PRKCD;FOS;KRT10;RAF1;ADCY7;FKBP5;CREB5
4 more..		
**Female Downregulated Genes (*n* = 305)**		
Ribosome	<0.001	RPL4;RPL5;RPL30;RPL41;RPL32;RPL12;RPL22;RPL11;RPL35A;MRPL36;MRPL24;RPL6;MR PL33;RPS25;RPL36AL;RPL35;RPL24;RPS20;RPL26;RPS27A;RPL39;RPS24;RPS12
Proteasome	<0.001	PSMB6;PSMA5;PSMB7;PSMA3;PSMD4;PSMC3;PSMC1;POMP;PSMB1;PSMC2;PSMD1;PSMF1
Spliceosome	<0.001	ISY1;HSPA8;SF3B5;CCDC12;BUD31;DDX42;PLRG1;PQBP1;SNRPD2;ZMAT2;SYF2;SNRPG;PP IH;SNRPA1;SNRPB2;SLU7;CTNNBL1
Protein export	<0.001	SRP19;SEC61G;SRPRB;SRP68;SRP14;SEC11A
Oxidative phosphorylation	<0.001	NDUFA9;NDUFA8;NDUFS5;COX17;NDUFB2;NDUFA1;COX6A1;ATP6V1E1;NDUFV2;COX6C;AT P6V1D;UQCRH
Huntington disease	<0.001	NDUFA9;NDUFA8;NDUFB2;NDUFA1;CLTA;COX6C;COX6A1;UQCRH;SOD1;SIN3A;NDUFS5;VD AC3;BAX;NDUFV2
Non-alcoholic fatty liver disease (NAFLD)	<0.001	NDUFA9;NDUFA8;NDUFS5;NDUFB2;NDUFA1;BAX;PIK3R1;COX6A1;NDUFV2;COX6C;ADIPO R2;UQCRH
Protein processing in endoplasmic reticulum	0.002	DNAJA1;ATXN3;HSPA8;HSP90AA1;HSPH1;HSP90AB1;EIF2AK1;SEC61G;ERP29;BAX;UBXN6
Parkinson disease	0.002	NDUFA9;NDUFA8;NDUFS5;VDAC3;NDUFB2;NDUFA1;COX6A1;NDUFV2;COX6C;UQCRH
Thermogenesis	0.007	NDUFA9;COA3;NDUFA8;SMARCC1;NDUFS5;COX17;NDUFB2;NDUFA1;COX6C;COX6A1; NDUFV2;UQCRH
3 more…		
**Male Upregulated Genes (*n* = 38)**		
**No enriched pathways**		
**Male Downregulated Genes (*n* = 50)**		
Proteasome	0.06	PSMD4;PSMC3;POMP

Similar to the brain analysis, we observed dramatically fewer enriched pathways in males with AD. Among upregulated genes in male AD patients, we did not identify any enriched pathways. Among downregulated genes in male AD patients, components of the proteasome were enriched including PSMD4 and PSMC3 (Table 3; Supplementary Table S13). For a full list of enriched pathways, refer to Supplementary Tables S11–S13.

Lastly, we performed a non-stratified analysis comparing gene expression between AD and control samples irrespective of sex in whole blood. Analyses were adjusted for sex, APOE ε4 carrier status, age and education. A total of 339 genes were upregulated and 360 genes were downregulated in patients with AD compared to controls (Supplementary Figure S4B, Supplementary Table S9). Upregulated genes were enriched for several pathways previously implicated in AD, including MAPK signaling, autophagy and NFkB signaling (Supplementary Table S15). In addition, a number of immune related pathways were enriched including tuberculosis, Escherichia coli infection, salmonella infection, and inflammatory bowel disease. Several components of the NFkB cascade and antigen presentation system including NFKBIA, ITGAM, STAT5B, TLR5, TLR4, CD14 and C4A, contributed to this enrichment (Supplementary Table S15). Among downregulated genes, pathways related to protein synthesis and metabolism, including ribosome, proteasome, protein export, thermogenesis, and oxidative phosphorylation were enriched. Included in these pathways were several oxidation phosphorylation related genes including NDUFA9, NDUFA8, COX4I2 (Supplementary Table S14).

### Network Analysis in Whole Blood Identifies a Stronger Disease Signature in Females

**FIGURE 4:**
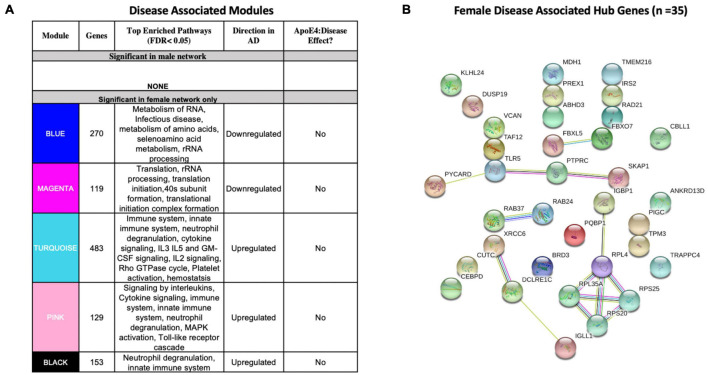
Network analysis in whole blood. WGCNA was used to construct gene network separately for males and females in whole blood. Networks were randomly assigned colors. **(A)** A description of the disease-associated gene networks (termed modules) produced using WGCNA. Significant disease-associated modules were identified by associating module eigengene to case/control status adjusting for age and APOE ε4 status and education (*P* < 0.05). KEGG enrichment analysis of significant was conducted using an adjusted *P-*value threshold of 0.05. The direction in AD is computed using the case/control coefficient of the model associating module eigengene to case/control status. Modules with significant APOE ε4 :disease interaction effect were identified by adding the interaction term APOE ε4 :disease to the previous model (*P* < 0.05). **(B)** Hub genes from female disease-associated modules. Hub genes were defined as genes with gene significance (the correlation between the gene expression and case/control status) greater than 0.2 and module membership (the correlation between gene expression and module eigengene) greater than 0.8. Hub genes were restricted to those that were differentially expressed in AD vs control. Protein-protein interactions between hub gene visualization was performed using the STRING v11 database. Edge color represents the type of interaction evidence for protein-protein interaction (cyan: known interaction from curated databases; turquoise: experimentally determined; green: gene-neighborhood predicted interaction; red: gene-fusions predicted interaction; blue: gene co-occurrence predicted interaction; green-yellow: text mining; black: co-expression; light purple: protein homology.

We identified five AD-associated modules in females and zero AD-associated modules in males (Figure 4) that met the significance threshold (FDR < 0.05) and were either positively or negatively correlated with case/control status. Among the modules in female samples, three modules including a 483-gene module (termed turquoise), a 129-gene module (termed pink) and 153-gene module (termed black) were upregulated in AD. Two modules including a 270-gene module (termed blue) and 119-gene module (termed magenta) were downregulated in AD (Figure 4A). No modules with significant APOE ε4:disease interaction effect were found in female or male network analyses from the blood datasets.

Enrichment analysis of disease-associated modules using the 2019 KEGG Human pathway database revealed pathways relevant to AD that were consistent with those identified in the single gene analysis (Figures 3A, 4A). For example, upregulated modules in females were strongly enriched for innate immune system activity, neutrophil degranulation, CSF signaling, IL2 signaling, and cytokine signaling. Consistent with single gene analyses, downregulated modules in females were enriched for metabolic processes including metabolism of RNA and metabolism of amino acids (Figure 4A).

There were 35 hub genes among disease associated modules in the female-specific network identified as module membership greater than 0.8, gene significance greater than 0.2 and differentially expressed between AD and controls (Figure 4B). In contrast, zero hub genes were identified in the male-specific gene network. Protein-protein interaction maps generated by STRING v11 suggest several interconnected genes including the B cell development related protein, IGLL1, and ribosomal proteins RPS20, RPS25, RPL4, and RPL35A (Figure 4B).

For a full list of genes in each module, including hub genes, please refer to Supplementary Tables S16–S17).

### Comparison of Brain and Blood Transcriptomic Signatures Reveals Common Immune Related Signals in Females

We next identified genes that were commonly dysregulated in both blood and brain (Figure 2E). In females, a total of 12 genes were dysregulated in the brain and blood in the same direction (all upregulated). Several genes among the commonly upregulated genes are known to be highly expressed in lymphoid tissue and play roles in immune cell recruitment including SERTAD1, ITGAX and TYROBP. In contrast, in males we found one upregulated gene, VCAN encoding vesican, dysregulated in both the blood and brain (Figure 2E).

### Cell-Type Deconvolution Identifies Sex-Specific Immune Cell Dysregulation in Females With AD

**FIGURE 5:**
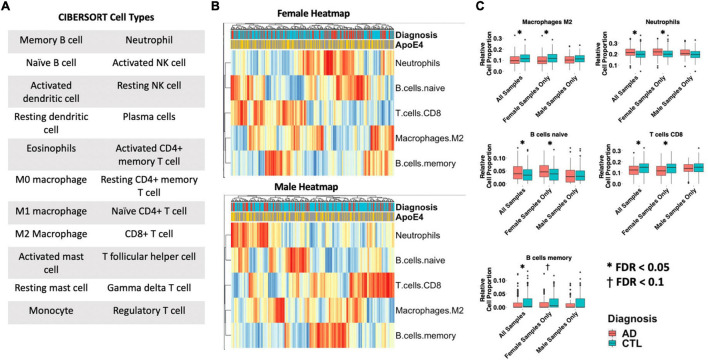
Cell type analysis in whole blood. **(A)** Cell types included in the panel of 22 reference cell types in CIBERSORT. **(B)** Heatmap depicting cell type expression between cases and controls. APOE ε4 carrier status, sex, and case/control status is annotated for each sample. Only cell types that are significantly different between cases and controls in pooled male and female, male-only or female-only analyses are shown. **(C)** Bar charts depicting cell type expression for individual cell types that are significantly between cases and controls in pooled male and female, male-only or female-only analyses are shown. Significance was assessed by associated cell type proportion to case/control status, adjusting for age, sex (in the pooled male and female model) and APOE ε4 status. *P* < 0.05 was deemed significant.

Differences in 22 immune blood cell types (Figures 5A,B) were evaluated by deconvolving the transcriptomic signature obtained via meta-analysis of blood studies. Analysis of cell type proportions adjusting for age, sex, and APOE ε4 status revealed an increase in neutrophils and naïve B cells, and a decrease in M2 macrophages and CD8+ T cells in AD patients compared to controls in pooled male and female samples (Figure 5C, FDR *P* < 0.05). Among females with AD, relative to controls, we observed an increase in neutrophils and naïve B cells and a decrease in M2 macrophages, memory B cells, and CD8+ T cells in AD samples (Figure 5C, FDR *P* < 0.05). Interestingly, among males with AD, we did not find any significant differences in immune cell proportions compared to controls.

### Sex-Specific Transcriptomic Data Improves AD Classification Accuracy

**FIGURE 6:**
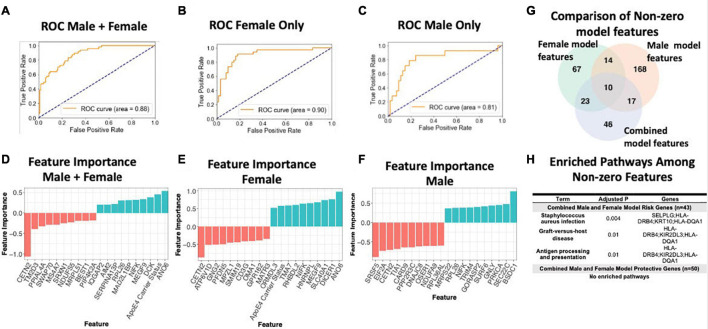
Linear SVM clinical + molecular model in whole blood. **(A–C)** Receiver operating characteristic (ROC) curves depicting performance of each linear SVM model on a test set composed of 25% of samples. Features include gene expression data obtained via meta-analysis, age, sex, education, and APOE ε4 status. Three models were fit for male and female pooled samples **(A)**, female samples only **(B)**, and male samples only **(C)**. **(D–F)** Feature importance plots for features with non-zero importance in the combined male and female model **(D)**, female model **(E)**, and male model **(F)**. A positive feature importance means that the expression of that feature increases the likelihood of being classified as AD (risk factor). A negative feature importance means that expression of the feature expression reduces the likelihood of being classified as AD (protective factor). **(G)** Comparison of non-zero features between combined male and female model, female model and male model. **(H)** Enriched pathways among non-zero features. An adjusted *P*-value cutoff of 0.05 was used for significance to increase power.

To assess the value of sex-specific transcriptomic data in developing a blood-based classifier in AD, we trained a linear SVM model to classify AD patients controls using the transcriptomic signature obtained via meta-analysis of blood studies. We trained a ‘clinical model’ with age, sex, education, and APOE ε4 status and a ‘clinical + molecular model’ with age, sex, education, APOE ε4 status, and blood transcriptomic data. Using pooled male and female samples, the ‘clinical + molecular model’ achieved a higher AUROC compared to the ‘clinical model’ (AUROC = 0.88 for ‘clinical + molecular model’; AUROC = 0.77 for ‘clinical model’) on a test set composed of 25% of samples (Figure 6A and Supplementary Figure S5A).

Interestingly, a model trained with only female data achieved a higher AUROC (‘clinical + molecular model’: 0.90 and ‘clinical model’: 0.86; Figure 6B and Supplementary Figure S5B) than the pooled male and female model. In contrast, a model trained with only male data obtained a lower AUROC (‘clinical + molecular’ model 0.81 and ‘clinical model’ 0.83; Figure 6C and Supplementary Figure S5C) than the pooled male and female model. These data suggest that including transcriptomic data improves disease classification in AD. Moreover, in the case of females, stratifying by sex improves upon a model using pooled male and female samples.

Figures 6G,H summarizes shared features between models. In all simple models (pooled male and female, female only, and male only), age and APOE ε4 status had a positive feature importance while education had a negative feature importance. A positive feature importance means that the expression of that feature increases the likelihood of being classified as AD (termed risk factor). A negative feature importance means that expression of the feature expression reduces the likelihood of being classified as AD (termed protective factor). In the female ‘clinical + molecular model,’ 58 features, including known risk factors including APOE ε4 and age, had a positive feature importance (Supplementary Table S18). In addition, 55 features had negative feature importance. Among these were education and previously implicated AD risk genes including CETN2 (Supplementary Table S18). In the male ‘clinical + molecular model,’ 104 features, including age, had positive feature importance (Supplementary Table S19). In addition, 105 features, including education, had negative feature importance (Supplementary Table S19).

Altogether, we observed a significant overlap (*P* < 0.001, hypergeometric test) in features with non-zero feature importance between the pooled male and female ‘clinical + molecular model’ and female ‘clinical + molecular model’; female ‘clinical + molecular model’ and male ‘clinical + molecular model’; and pooled male and female ‘clinical + molecular model’ and male ‘clinical + molecular model’ (Figure 6G).

Functional annotation of features with a non-zero feature importance was performed via enrichment analysis using the 2019 KEGG database of human pathways. Among features with non-zero feature importance, we did not identify any enriched biological pathways in the male only and female only complex models. In the male and female pooled complex model, features with positive feature importance (risk factors), were enriched for staphylococcus aureus infection, graft-vs-host disease, and antigen presentation and processing KEGG pathways (adjusted *P* < 0.05; Figure 6H). The HLA genes HLA-DRB4 and HLA-DQA1 contributed to this enrichment. In addition, the P-selection glycoprotein ligand-1 gene (SELPLG) and killer cell immunoglobulin-like receptor (KIR2DL3) also contributed to enrichment, suggesting a role for leukocyte recruitment and natural killer cell activity in AD pathology.

To answer the question whether transcriptomic data has predictive value independent of clinical features such as age and education, we conducted two additional analyses. Specifically, we first built three models with only the transcriptomic data as features, (1) pooled male and female samples, (2) male samples only, and (3) female samples only. In predicting AD status, these models performed with a AUROC of 0.85, 0.90, 0.73 for the pooled male and female model, female samples only and male samples only, respectively (Supplementary Figure S6). We next used the ComBat function in R to adjust the original transcriptomic data for ApoE4 status, years of education and age. We then built three models using only this adjusted transcriptomic data as features: (1) pooled male and female samples, (2) male samples only, and (3) female samples only. In these models, we would expect similar performance compared to the clinical+molecular model reported in the original manuscript. As expected, these models performed similarly to the clinical+molecular models with an AUROC of 0.89, 0.90, 0.81 for the pooled male and female model, female samples only and male samples only, respectively (Supplementary Figure S6). Given the well-established role of age, sex, ApoE4 and education in AD, it is not surprising that clinical only models perform well. However, our analyses suggest that transcriptomic data has predictive power independent of clinical data.

### Down Sampling Sensitivity Analysis

Supplementary Figure S7 describes the analytical approach for down sampling our blood and brain datasets. Specifically, we performed 100 iterations in which we down sampled the female dataset such that the total number of female AD cases and controls was equal to the number of male AD cases and controls. In each iteration, we performed sex-stratified differential expression and computed the number of AD-associated genes and derived a 95% confidence interval. We randomly selected one iteration to replicate the functional analyses, network analyses, cell-type deconvolution and machine learning analyses as found in the analysis without down sampling.

Differential expression results in the brain revealed a significantly higher mean number of differentially expressed AD genes in females compared to males (*p* < 0.01), consistent with our original findings (Supplementary Table S21). Similarly, in the blood, differential expression results in blood revealed a greater than 3-fold increase in the number of differentially expressed AD genes in females compared to males (*p* < 0.001), consistent with our findings from the analysis without down sampling (Supplementary Table S22).

We next selected one down sampled iteration for follow up evaluation of enriched pathways in genes differentially expressed between AD cases and controls. In both the randomly selected iterations from blood and the brain, we were able to replicate nearly every enriched pathway observed in the entire dataset. Unless otherwise indicated, Supplementary Figure S8 displays the top 5 enriched pathways (adjusted *P* < 0.05) in each group of genes (i.e., male upregulated in AD, female upregulated in AD, etc.).

To assess whether network changes observed in the entire dataset are preserved in the down sampled dataset, we selected the same iteration described previously to perform Weighted Gene Network Correlation Analysis (WGCNA). Consistent with original analysis, we created a WGCNA network separately in males and females to derive modules (or groups of genes) within sex-stratified data. Because only the female dataset was down sampled, module preservation between the down sampled dataset and entire dataset was computed only for the female dataset. In the brain, we found 10 modules, each with *Z*-summary score > 10 suggesting strong preservation in the entire dataset (Supplementary Figure S9A). Similarly, in blood, we found 11 modules, each with a *Z-*Summary score greater than 10, 10 suggesting strong preservation in the entire dataset (Supplementary Figure S9B). Overall, this analysis suggests that network effects in the down sampled dataset are strongly preserved in the entire dataset.

To assess whether the cell type deconvolution results are replicated in the down sampled dataset, we selected the same iteration described previously and computed cell type proportions using CIBERSORT. Supplementary Figure S10 results are presented for both the entire dataset (B) and down sampled dataset (C). In the down sampled dataset, we observed that levels of M2 macrophages, neutrophils, naïve B cells, CD8 T cells, memory B cells were significantly different between AD cases and controls among females (*p* < 0.05, Supplementary Figure S10C). Upon pooling both male and female samples we similarly observed dysregulation in M2 macrophages, neutrophils, naïve B cells, CD8 T cells, memory B cells. We did not observe dysregulation in any of the CIBERSORT cell types (Supplementary Figure S10A) among male samples (Supplementary Figure S10C). These results of cell type deconvolution with the down sampled dataset (Supplementary Figure S10C) are consistent with the cell type changes we observed in the entire dataset (Supplementary Figure S10B).

To assess whether the performance of a linear support vector machine (SVM) model with l1 regularization used to classify AD cases and controls based on blood gene expression data was different in the down sampled data compared to the entire dataset, we created receiver operating characteristic (ROC) curves depicting performance of each linear SVM model on a test set composed of 25% of samples. Features include gene expression data obtained via meta-analysis, age, sex, education, and APOE ε4 status. Models were fit for female samples only (Supplementary Figures S11A,C), and male samples only (Supplementary Figures S11B,D). While we did not down sample the male dataset, the performance in the male dataset was slightly different compared to the original manuscript (AUROC = 0.80 vs AUROC = 0.81 in the original dataset). This difference can be ascribed to using a random seed when training the SVM. In the down sampled dataset, consistent with our original claims (Supplementary Figures S11A,B), we observed a higher AUROC in a model trained on female samples (AUROC = 0.85; C) compared to a model trained on male samples (AUROC = 0.80; Supplementary Figure S11D). Overall, these results suggest that performance differences in male and female samples are not strongly driven by sample size differences.

## Discussion

In this study, through computational analysis of publicly available gene expression datasets from brain and blood samples, we evaluated AD at the transcriptome level using single gene and network approaches to gain insight into the mechanisms underlying sex and APOE ε4 - genotype based differences in AD. We also evaluated how including sex-specific transcriptomic data from blood samples with clinical data would affect the performance of a machine learning classifier for AD diagnostics. In addition to identifying putative immune-related pathways for further analysis, we recapitulate known processes in AD including the downregulation of metabolic pathways including oxidative phosphorylation and the TCA cycle (Ciryam et al., 2016; Kundra et al., 2017).

Our characterization of brain transcriptomic signatures revealed, among upregulated genes in the brains of both females and males with AD, an enrichment of pathways related to components of the innate and adaptive immune systems as well as the MAPK signaling pathway. This result is consistent with past findings where the brain’s immune system has been indicated as a major component of AD pathogenesis (Heneka et al., 2015; Frost et al., 2019). Additionally, MAPKs, enzymes that play critical roles in cellular signaling, have also been implicated as accelerators of AD development (Du et al., 2019). Overall, findings from our brain transcriptome analysis provide supporting evidence for therapeutics currently being explored for AD, such as p38 MAPK inhibitors (Lee and Kim, 2017), and suggest that possible treatments targeting the MAPK pathway may have a greater effect in females with AD.

Interestingly, from our differential expression analysis, we found a 30% greater number of dysregulated genes in the brain transcriptome that met our significance cutoff for females with AD compared to males with AD (477 vs 366, respectively). Many of these genes are in pathways related to antigen presentation and processing, complement activation, suggesting a female-specific role of neuroinflammation in the pathogenesis of AD. Additionally, for downregulated genes in AD patients, we observed enrichment of neurological signaling pathways in females only and no enriched pathways in males.

Through network analysis, we identified more AD-associated modules in the brain transcriptome of females than males. Enrichment analysis of AD-associated modules also revealed some pathways that were enriched in both sexes, including an upregulated module for a PI3/Akt signaling related pathway and downregulated modules for oxidative phosphorylation and thermogenesis related pathways. Unique to females, we observed upregulated modules associated with cell structural processes (adherens junctions, actin cytoskeleton and axonal guidance) and HSV infection-related zinc finger nuclease genes, as well as a downregulated module for neurological signaling pathways, autophagy and proteolysis. Compared to single gene analysis, network-based approaches have the benefit of identifying biologically relevant programs of correlated gene expression comprised of many genes, whose individual expression changes may be small. In addition, network-based approach overcomes the multiple hypothesis correction issue by grouping genes into co-expression modules first prior to association analysis. While one may expect the result of single gene and network approaches to be correlated, we do not expect them to be identical. For example, in both the female and male brain analyses, downregulated modules were enriched for oxidative phosphorylation related pathways consistent with existing work (Ciryam et al., 2016). However, in the single-gene analyses, we were unable to recapitulate these oxidative phosphorylation-related pathways.

Upon performing hub gene analysis, we identified hub genes in female disease-associated modules but were unable to identify male disease associated hub genes. These female hub genes consisted of several potentially interconnected genes including ITPKB, PDGFRB, GNG12, and GNA12. In our subsequent analysis to assess an APOE ε4 :disease interaction effect, we identified three modules, one of which was significantly enriched for HSV infection-related zinc finger nuclease genes as well as containing the ITPKB hub gene as a highly connected regulator. These results suggest zinc finger nucleases as a potential mechanism underlying sex-associated differential penetrance of APOE ε4 in AD.

Our findings suggest a neuroinflammatory model of AD pathogenesis in females with dysregulation in components of the adaptive and innate immune system including antigen presentation and processing and complement activation and genes including MAPK and ITPKB. It has been postulated that accumulation of damage from HSV infection and major neuroinflammatory effects can lead to the development of AD, and that APOE ε4 carriers suffer either greater viral damage or have poorer repair of such damage (Itzhaki, 2018). Previous studies have demonstrated that ITPKB expression is increased in human AD brains and exacerbates AD pathology in an animal model (Stygelbout et al., 2014). Our brain transcriptome findings for females with AD, including downregulation of autophagy and proteolysis pathways, upregulation of pathways related to the immune system and HSV infection, as well as ITPKB as a hub gene, particularly in female APOE ε4 carriers, highlight specific gene-encoded processes in the brain that may be more involved in AD for women than for men.

Similar to our brain findings, in analysis of blood transcriptomes, we observed more dysregulated genes in the blood of females with AD than in males with AD. Further characterization of these transcriptomic signatures revealed, among upregulated genes, enrichment in only females with AD of pathways related to components of the innate and adaptive immune systems as well as actin cytoskeleton regulation; however, for downregulated AD genes, we observed enriched metabolic pathways (oxidative phosphorylation and thermogenesis) in females and enriched pathways for protein homeostasis in males.

Through network analysis, we identified AD-associated modules and hub genes in the female blood transcriptome but not in males. In the blood of females with AD, upregulated modules were strongly enriched for innate immune system activity (neutrophil degranulation, CSF signaling, IL2 signaling, and cytokine signaling). Consistent with single gene analyses, female downregulated modules were enriched for metabolic processes (e.g., metabolism of RNA and amino acids). Hub genes identified in the blood of females with AD include those related to immunity (the B cell development related protein, IGLL1) and viral RNA translation (ribosomal proteins RPS20, RPS25, RPL4, and RPL35A).

In addition to neuroinflammation’s role in AD, dysregulation of the immune system outside of the brain has also been noted to be a factor in AD (Cao and Zheng, 2018). Our findings feature specific gene-encoded processes in peripheral blood cells that may be more involved in AD for women than for men. Furthermore, our cell-type deconvolution analysis revealed dysregulation of peripheral immune cells uniquely in females with AD and not males with AD.

When including blood transcriptomic features with clinical features (age, sex, education, and APOE ε4 status) to train a machine learning prediction model of AD, our model performed better with these additional molecular features than without (AUROC: 0.88 vs 0.77, respectively). The performance of this model also improved when trained with only female data (clinical + molecular model AUROC: 0.90 and clinical model AUROC: 0.86) and worsened when trained with only male data (clinical + molecular model AUROC: 0.81 and clinical model AUROC: 0.83) than with pooled male and female model. This finding suggests that the molecular changes in females compared to males are better able to model AD-related changes. Further, given the distinct transcriptomic signature observed in males and females, stratifying by sex may aid future efforts to identify biomarkers in AD.

Diagnostic tests currently available for AD, including Aβ position emission tomography (PET), lack accuracy or are implemented through invasive and painful procedures such as lumbar puncture (Ewers et al., 2015; Elahi and Miller, 2017; Ritchie et al., 2017; Bergeron et al., 2018). Diagnostic tests for AD that are more accurate and less invasive are worthwhile for preventing undue uncertainty and physical discomfort experienced by patients. Our machine learning AD prediction model based on clinical and blood transcriptomic features has the potential to complement currently available clinical AD diagnostic tests, and improve the accuracy of these tests, particularly for women, with minimal additional discomfort for patients.

## Limitations

Based on the nature of our analyses, there are a number of limitations to note. First, an inherent limitation of retrospective transcriptomic analyses is the ability to generate mechanistic hypotheses rather than establish causality. While previous studies have investigated sex-specific transcriptomic signatures in AD, this is the first study to integrate network, single gene analytic and machine learning approaches (Wan et al., 2020). We analyzed publicly available datasets, which were limited in sample size and contained annotation differences. This provided challenges in selecting cases from controls and restricted our ability to answer certain questions. For instance, the Allen Brain Atlas dataset provided only a binary classification for apoE (APOE ε4 : Y/N). This confined our analysis to only look at the presence of APOE ε4, instead of looking at difference across different genotype combinations. Next, we did not stratify our analysis by age or disease stage, so we cannot describe whether these transcriptomic signatures differ with age or disease severity. It is possible that transcriptomic differences observed in our study could be explained partially by the effect of analgesics, anti-inflammatory medications or acetylcholinesterase inhibitors. The lack of medication information prevented us from controlling for the effects of these medications. Further, our brain and blood transcriptomic data were profiled using different technologies (RNA-sequencing and microarray). While we expect results from each technology to be correlated, systemic biases of each platform may limit direct comparison between brain and blood results. Additionally, since we aggregated bulk tissue from different brain regions in our analysis, we cannot infer sex differences across brain region. Amyloid and tau pathology are known to vary across brain regions, and as such our results may be partially driven by differential amyloid and tau burdens. Given this study’s use of public datasets, we were not able to address the degree to which amyloid and tau pathology affect the sex-specific gene expression changes we observed. Consequently, using bulk tissue transcriptomics reduces our resolution of the more complex interactions and contributions of different brain cell types in AD. It is possible that differences in the proportion of certain cell types, rather than broad gene expression changes, could explain the changes observed in our study. Future approaches to better characterize sex-specific changes in AD would involve stratification by brain regions, age and disease stage, apoE genotype, as well as an analysis of single cell AD datasets. Interestingly, a recent study (Phongpreecha et al., 2020) used single-cell approaches to quantify activation of 15 intracellular signaling pathway activation in peripheral blood mononuclear cells between individuals with AD and healthy controls. This study found increased activation of the STAT5 pathway in AD, consistent with our results demonstrating increased STAT5B in the blood of females with AD compared with healthy females.

Another limitation may stem from the fact that in both our brain and blood transcriptomic data, the ratio of female AD cases:controls was greater than the ratio of male AD cases:controls. To assess whether our results are primarily driven by a difference in statistical power between males and females, we performed 100 iterations of down sampling. In each iteration, we down sampled our dataset such that the ratio of AD cases:controls was the same in the male and female groups. We then repeated the analyses performed on the original dataset without down sampling. This ensures that our results are not driven by differences in statistical power between males and females. Subsampling analysis revealed a strong preservation of our original findings, suggesting that our results are not strongly influenced from a mismatch in sample size between males and females.

## Conclusion

In conclusion, the major finding of this study is a distinct, sex-specific transcriptomic signature in the brains and whole blood of patients with AD. Gene expression meta-analysis and network-based analyses revealed an immune signature in the brains and whole blood of females with AD that was absent in males. Our analyses also revealed more pronounced neurosignaling and metabolism signatures in the brains whole blood of females with AD than in males with AD. Stratification by sex improved machine-learned based classification of AD using whole-blood transcriptomic data. Results from this work will help to better understand molecular etiologies underlying sex differences in AD and pave the way for sex-specific biomarker and therapeutic development in AD.

## The Members of the Alzheimer’s Disease Neuroimaging Initiative

Data collection and sharing for this project was funded by the Alzheimer’s Disease Neuroimaging Initiative (ADNI) (National Institutes of Health Grant U01 AG024904) and DOD ADNI (Department of Defense award number W81XWH-12-2-0012). ADNI is funded by the National Institute on Aging, the National Institute of Biomedical Imaging and Bioengineering, and through generous contributions from the following: AbbVie, Alzheimer’s Association; Alzheimer’s Drug Discovery Foundation; Araclon Biotech; BioClinica, Inc.; Biogen; Bristol-Myers Squibb Company; CereSpir, Inc.; Cogstate; Eisai Inc.; Elan Pharmaceuticals, Inc.; Eli Lilly and Company; EuroImmun; F. Hoffmann-La Roche Ltd and its affiliated company Genentech, Inc.; Fujirebio; GE Healthcare; IXICO Ltd.; Janssen Alzheimer Immunotherapy Research & Development, LLC.; Johnson & Johnson Pharmaceutical Research & Development LLC.; Lumosity; Lundbeck; Merck & Co., Inc.; Meso Scale Diagnostics, LLC.; NeuroRx Research; Neurotrack Technologies; Novartis Pharmaceuticals Corporation; Pfizer Inc.; Piramal Imaging; Servier; Takeda Pharmaceutical Company; and Transition Therapeutics. The Canadian Institutes of Health Research is providing funds to support ADNI clinical sites in Canada. Private sector contributions are facilitated by the Foundation for the National Institutes of Health (www.fnih.org). The grantee organization is the Northern California Institute for Research and Education, and the study is coordinated by the Alzheimer’s Therapeutic Research Institute at the University of Southern California. ADNI data are disseminated by the Laboratory for Neuro Imaging at the University of Southern California.

## Data Availability Statement

The original contributions presented in the study are mentioned in the article/Supplementary Material, further inquiries can be directed to the corresponding authors.

## Ethics Statement

Ethical review and approval was not required for the study on human participants in accordance with the local legislation and institutional requirements. The patients/participants provided their written informed consent to participate in this study.

## Author Contributions

MP and MS conceived and designed the study. MP, AT, and BG extracted data from publicly available data sources used in the study. MP, JW, IP, and AG completed all analyses and produced figures. MP, MS, TO, and SB wrote the manuscript. MP, MS, TO, SB, AT, KZ, BG, and YH critically revised the manuscript for important intellectual content. All authors commented and approved the manuscript.

## Conflict of Interest

YH is a co-founder and scientific advisory board member of E-Scape Bio, Inc. and GABAeron, Inc. MS is on the scientific advisory board for Aria Pharmaceuticals. The remaining authors declare that the research was conducted in the absence of any commercial or financial relationships that could be construed as a potential conflict of interest.

## Publisher’s Note

All claims expressed in this article are solely those of the authors and do not necessarily represent those of their affiliated organizations, or those of the publisher, the editors and the reviewers. Any product that may be evaluated in this article, or claim that may be made by its manufacturer, is not guaranteed or endorsed by the publisher.

## Footnotes

1
adni.loni.usc.edu

## Abbreviations

A β: amyloid β
AD: Alzheimer’s disease
APOE ε 4: apolipoprotein E ε 4 allele
GEO: Gene Expression Omnibus
MSBB: Mount Sinai Brain Bank
RNA-Seq: RNA-sequencing
ROC: receiver operating characteristic
ROSMAP: Religious Orders Study and Memory and Aging Project.
