# Supplementary material for: Sex-Specific Cross Tissue Meta-Analysis Identifies Immune Dysregulation in Women With Alzheimer’s Disease
Source: Front Aging Neurosci. 2021 Sep 30;13:735611. doi: 10.3389/fnagi.2021.735611 (PMC8515049; doi:10.3389/fnagi.2021.735611)
Supplement: Supplementary file 2 [file Data_Sheet_1.DOCX]

**Supplementary Figures**


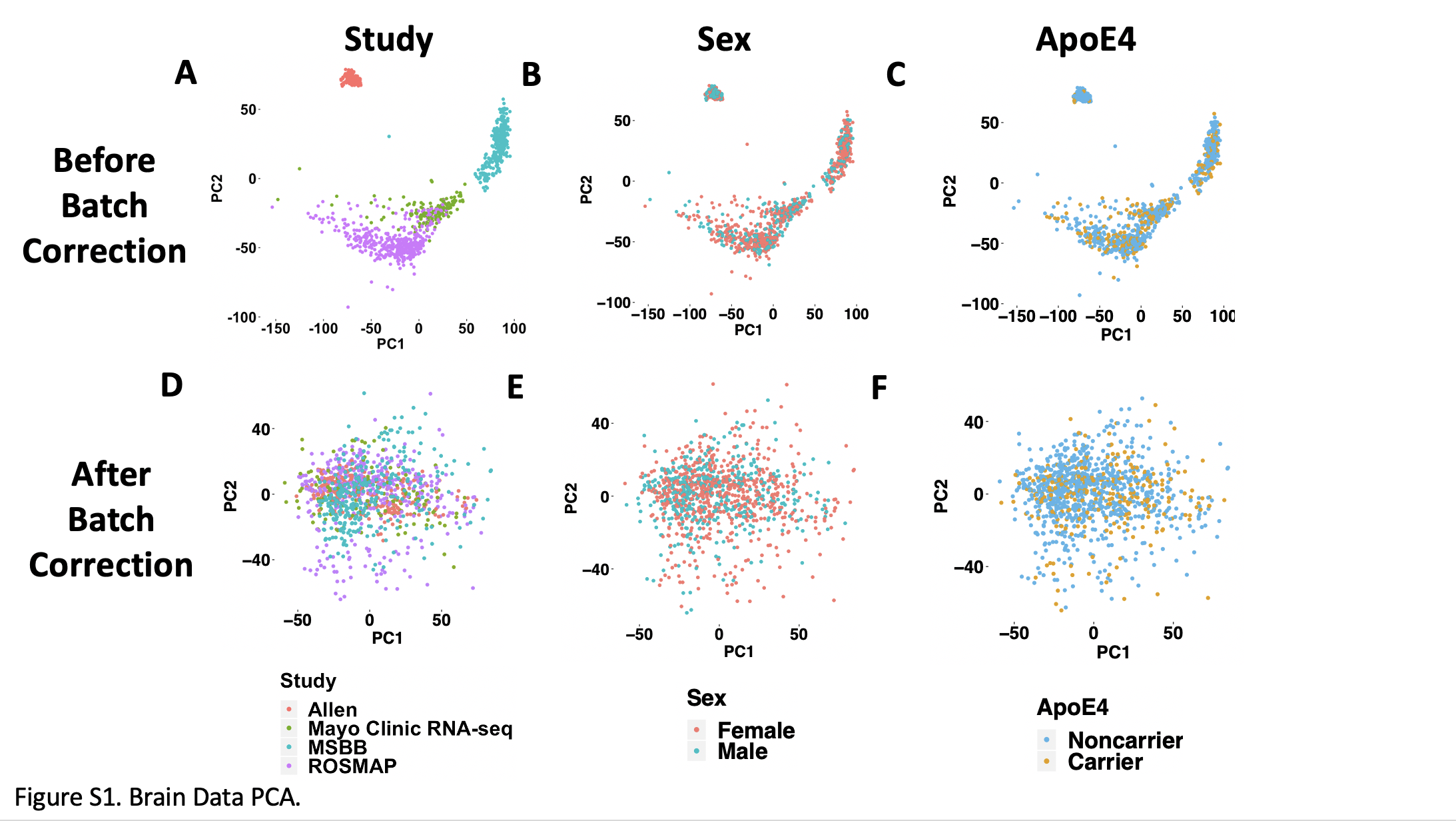


**Figure S1: Brain Data PCA**

Description: Principal component plots (PCA) of brain samples before (top) and after batch (bottom) correction. PCA plots depict principal component 1 and 2 and are colored by study (left), sex (middle) and APOE ε4 status (right).


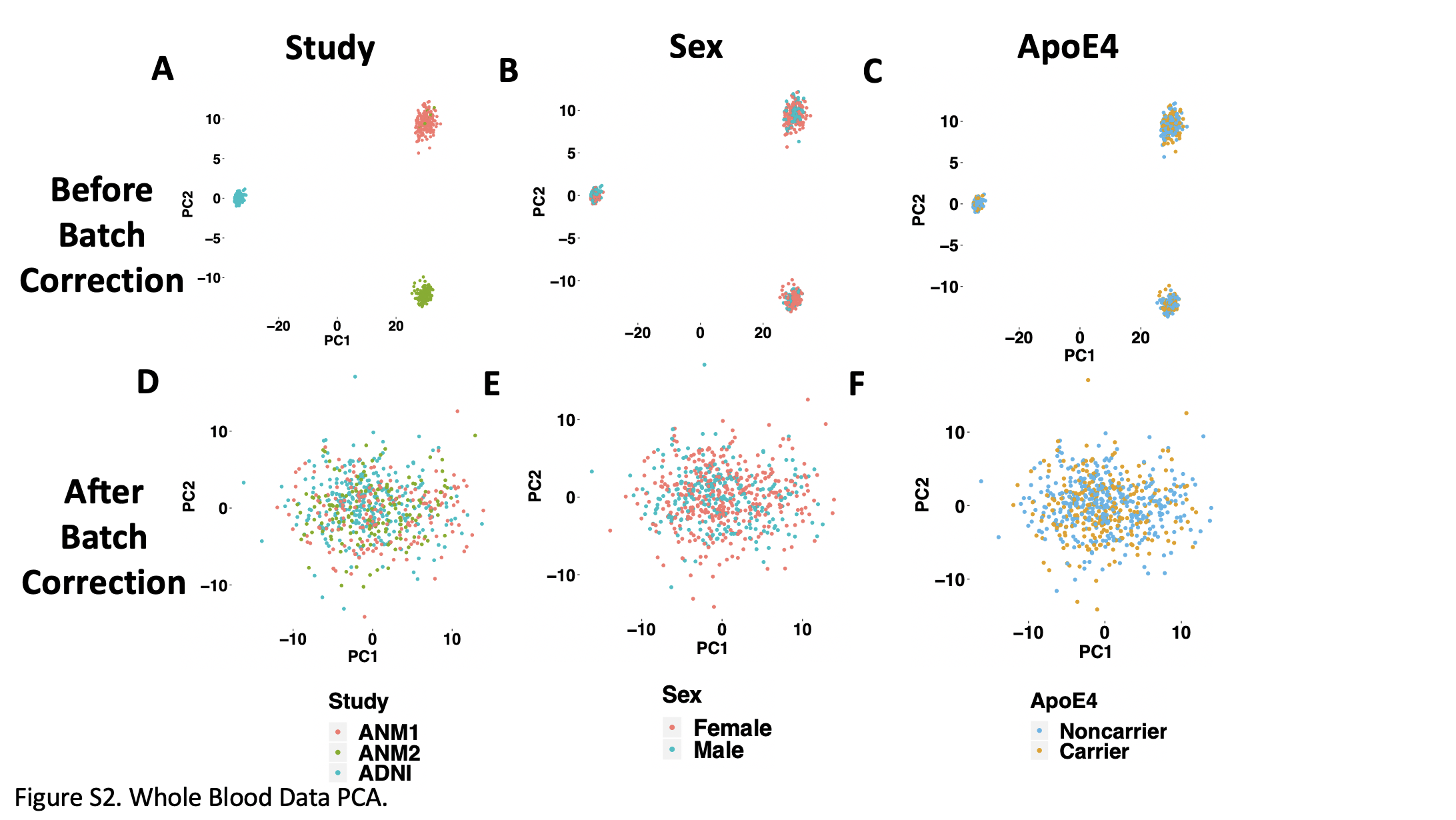


**Figure S2: Blood Data PCA**

Description: Principal component plots (PCA) of blood samples before (top) and after batch (bottom) correction. PCA plots depict principal component 1 and 2 and are colored by study (left), sex (middle) and APOE ε4 status (right).


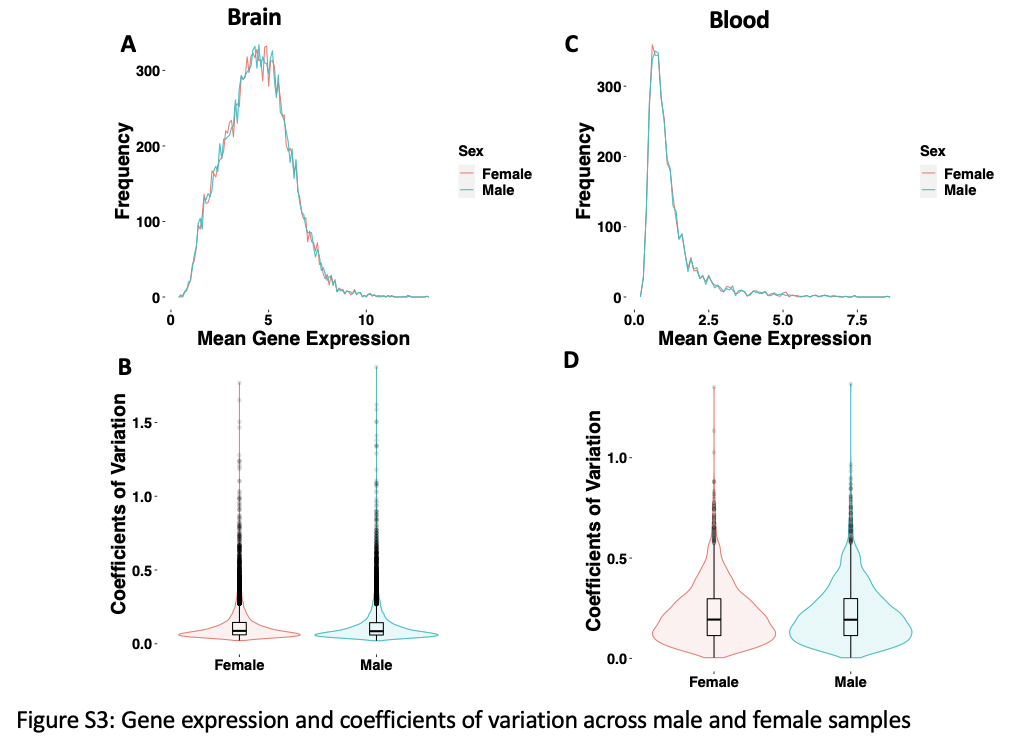


**Figure S3: Gene expression and coefficients of variation across female and male samples**

Description: **A, C** Distribution of mean gene expression across male and female samples in the brain (**A**) and blood (**C**) datasets. **B, D** Violin plots depicting coefficients of variation for each gene across the male and female samples in the brain (**B)** and blood (**D**) datasets.


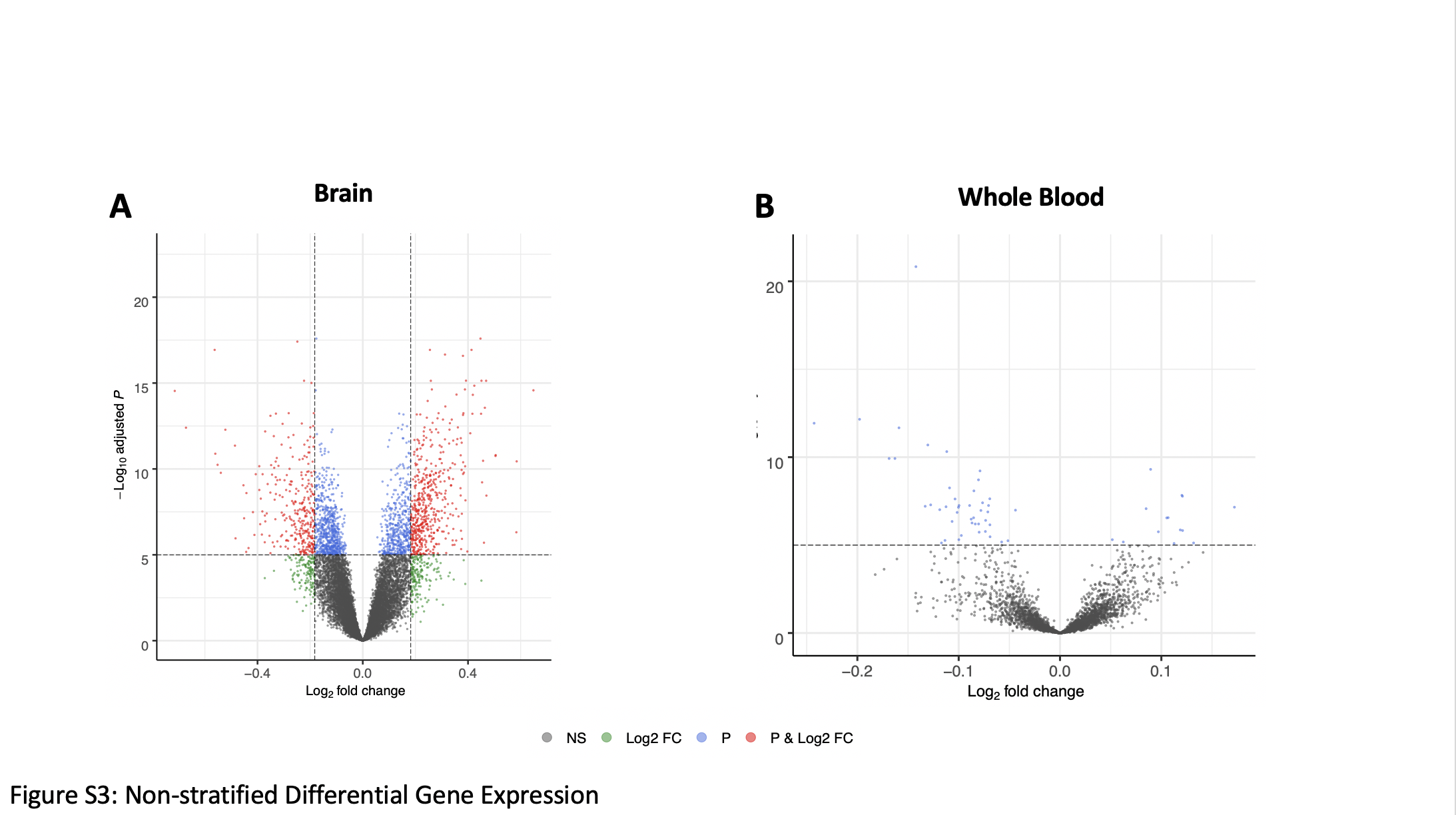
**Figure S4**: **Non-stratified Differential Gene Expression**

Description: **A.** Volcano plot depicting fold changes and p values from samples in the brain. Analyses were adjusted for sex, APOE ε4 status, and age. An adjusted *P* value < 0.05 and FC > 1.2 was deemed significant. In the brain, a total of 662 genes were upregulated and 430 genes were downregulated in patients with AD compared to controls. **B.** Volcano plot depicting fold changes and *P* values from samples in the blood. Analyses were adjusted for sex, APOE ε4 status, age, and education. An adjusted *P* value < 0.05 was deemed significant. In blood, 339 genes were upregulated and 360 genes were downregulated in patients with AD compared to controls.


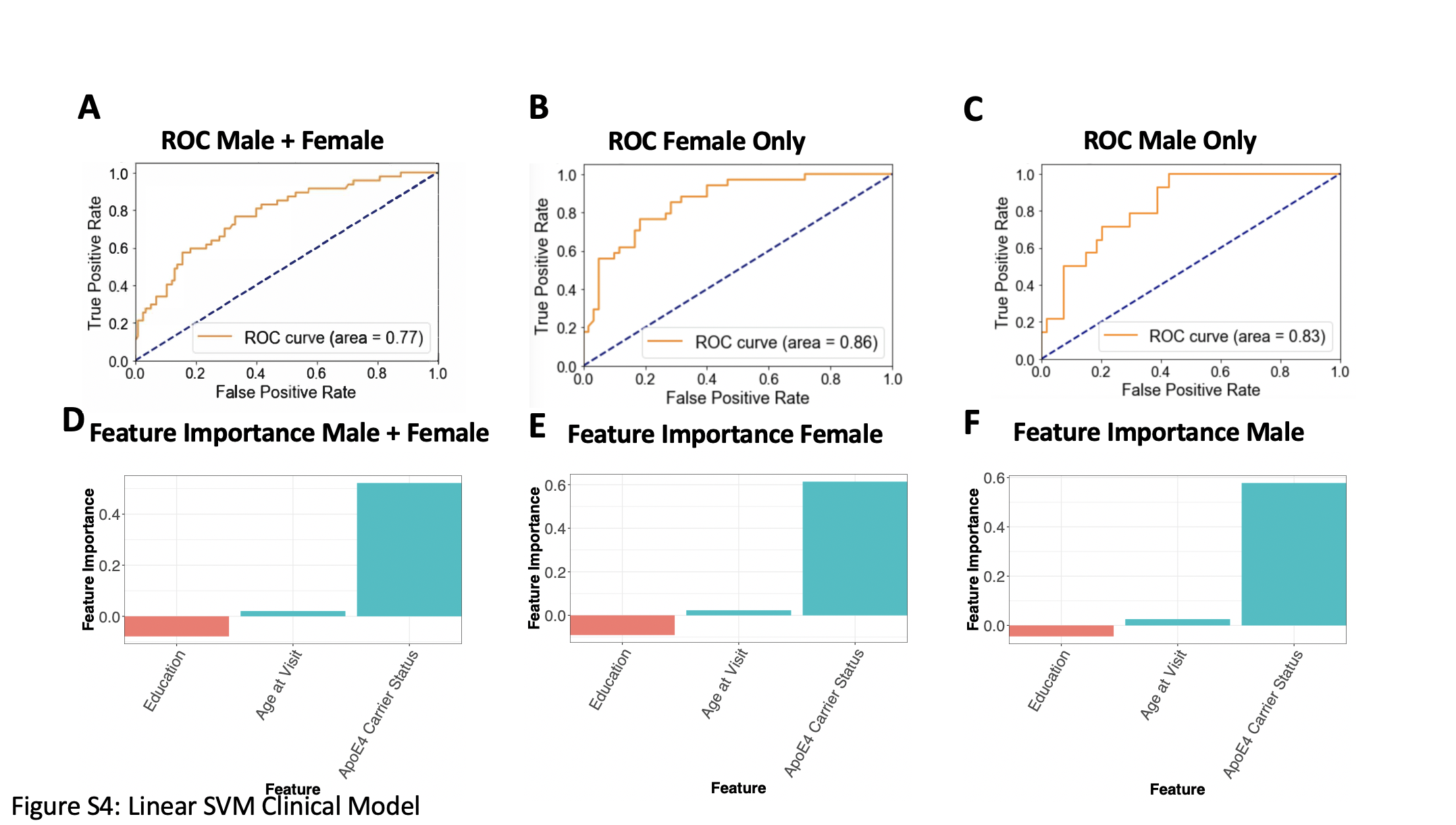
**Figure S5: Linear SVM Clinical Model**

Description: **A-C.** Receiver operating characteristic (ROC) curves depicting performance of each linear SVM model on a test set composed of 25% of samples. Features include age, sex, education, and APOE ε4 status. Three models were fit for male and female pooled samples (**A)**, female samples only (**B**), and male samples only (**C**). **D-F.** Feature importance plots for features with non-zero importance in the combined male and female model (**D)**, female model (**E**), and male model (**F**). A positive feature importance means that the expression of that feature increases the likelihood of being classified as AD (risk factor). A negative feature importance means that expression of the feature expression reduces the likelihood of being classified as AD (protective factor).


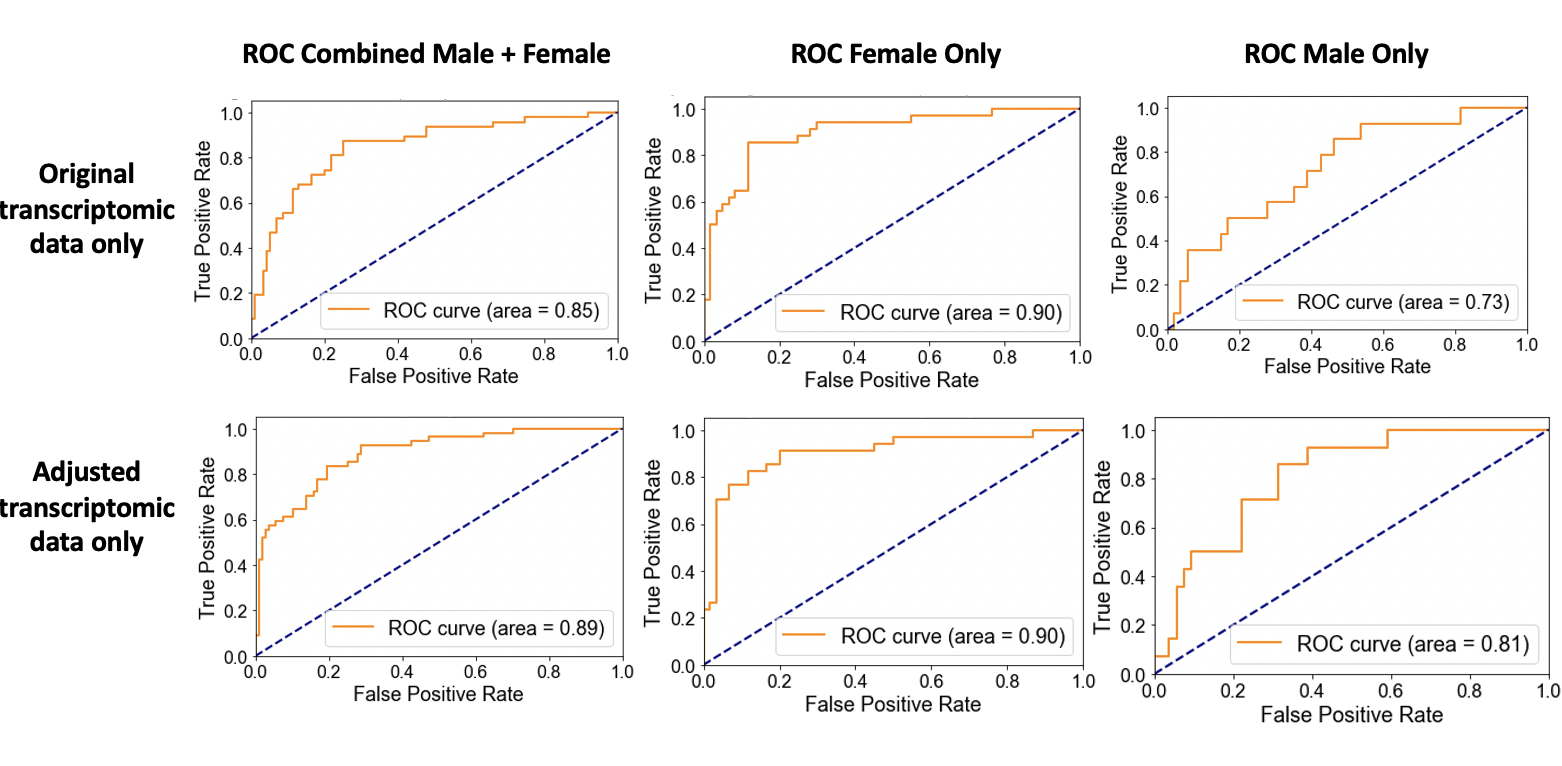


**Figure S6: Linear SVM performance using transcriptomic data only**

Receiver operating characteristic (ROC) curves depicting performance of each linear SVM model on a test set composed of 25% of samples. To assess the predictive power of gene expression independent of clinical data, each linear SVM model was trained using gene expression only. Features include gene expression data, unadjusted for age, sex, ApoE4 status, and education (**top row**) or gene expression data that was adjusted for age, sex, ApoE4 status, and education (**top row**) using the ComBat package in R. Three models were fit for male and female pooled samples (**right)**, female samples only (**middle**), and male samples only (**left**).


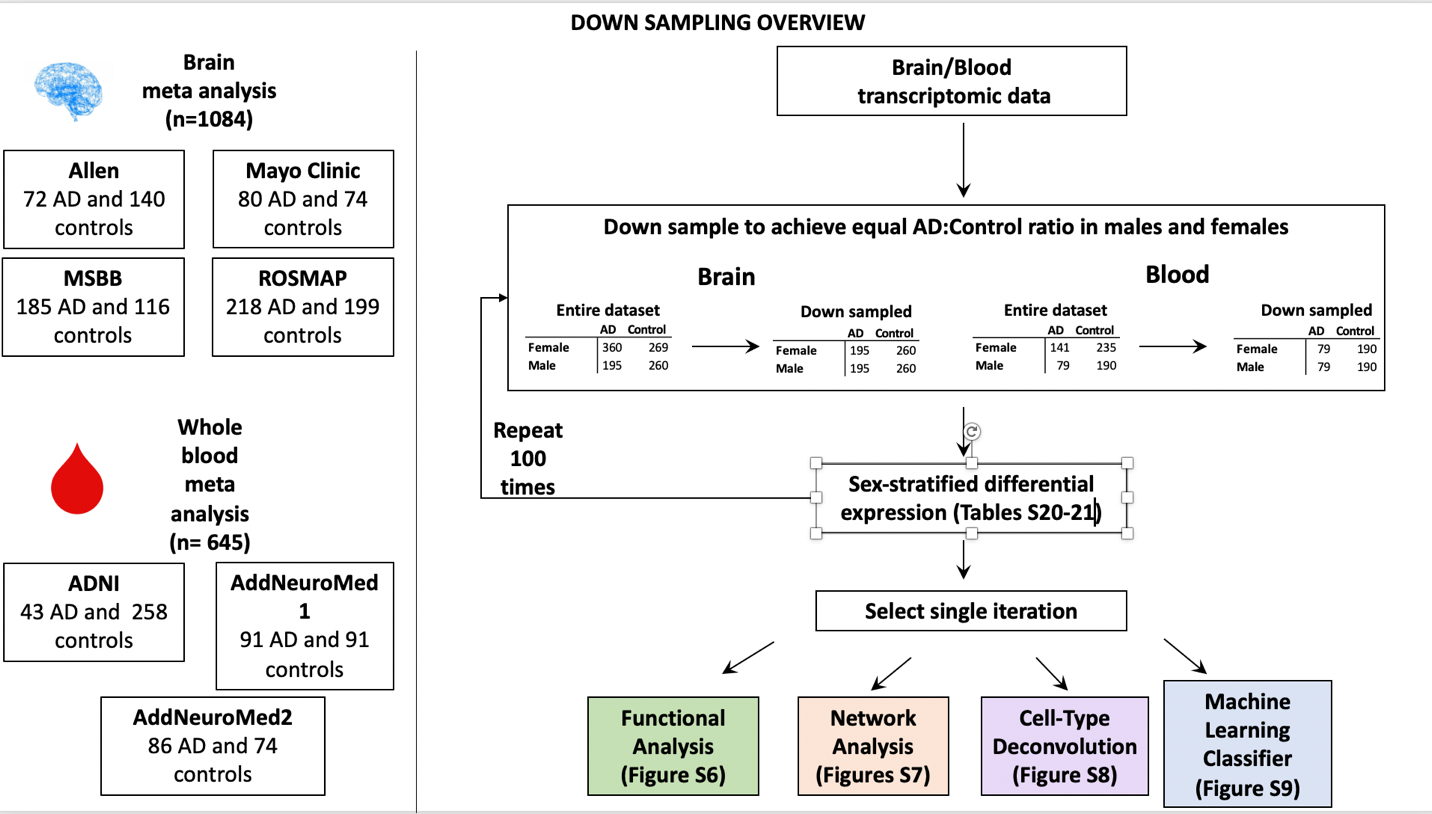
**Figure S7: Down Sampling Overview**

Diagram depicting the additional analyses performed as part of the downsampling sensitivity analyses. To assess whether our results are primarily driven by a difference in statistical power between males and females, we performed 100 iterations of down sampling. In each iteration, we down sampled our dataset such that the ratio of AD cases:controls was the same in the male and female groups. We then repeated the analyses in the original manuscript, including functional analysis, differential expression, network analyses, cell type deconvolution analyses and machine learning classification.

**Figure S8: Enriched pathways in down sampled dataset**

Description: KEGG 2019 Human pathways significantly enriched among differentially expressed genes in the down sampled dataset are presented. Pathways represent an adjusted P value < 0.05. For each category, the top 5 enriched pathways are presented.


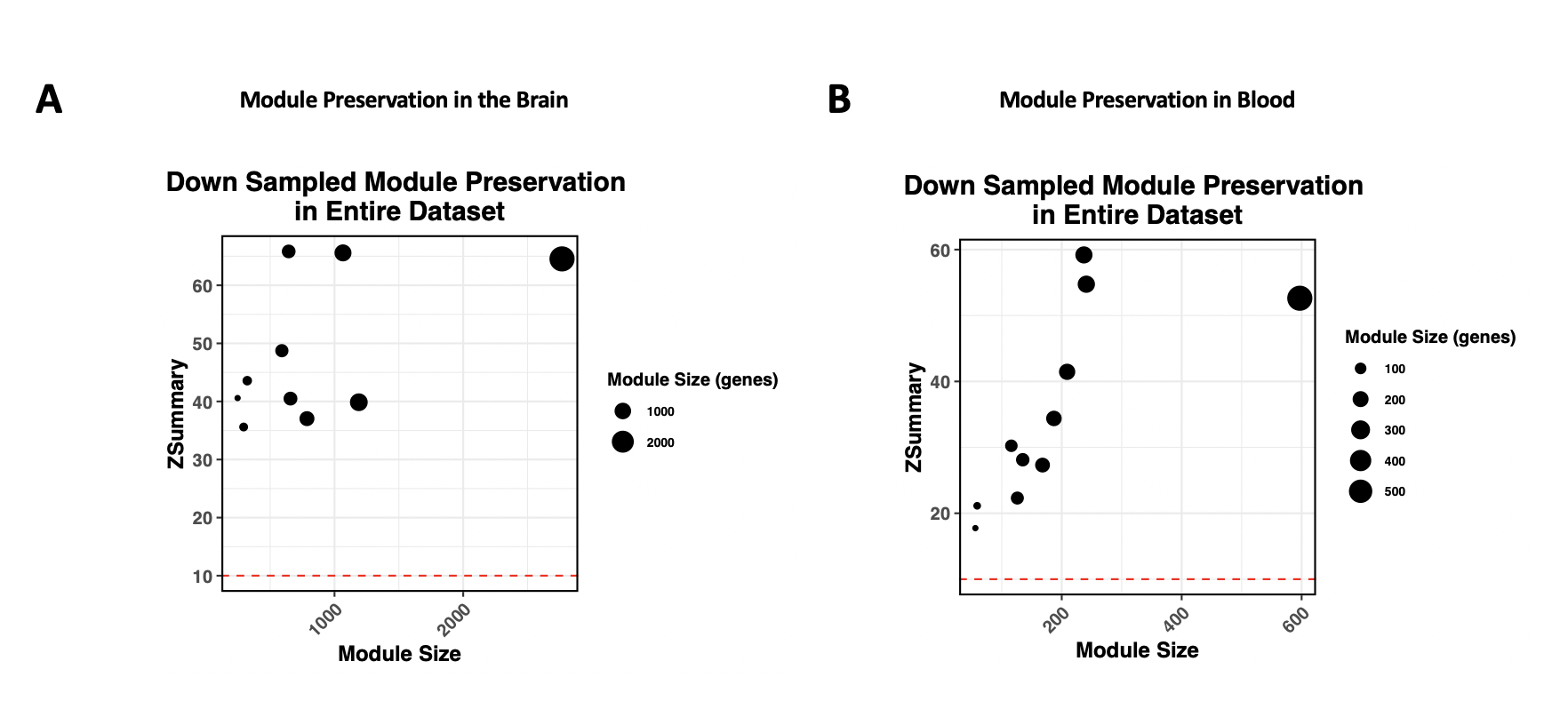
**Figure S9: Network analysis in down sampled dataset**

Description: To assess whether network changes observed in the entire dataset are preserved in the down sampled dataset, we selected the same iteration in described previously to perform WGCNA analysis. Consistent with original analysis, we created a WGCNA network separately in males and females to derive modules (or groups of genes) within sex-stratified data. Because only the female dataset was down sampled, module preservation between the down sampled dataset and entire dataset was computed only for the female dataset. Module preservation was computed as described in the original manuscript to derive a ZSummary value for each module. A Zsummary value > 10 indicates evidence of strong preservation. Module Zsummary score is plotted against module size (number of genes). All modules in the down sampled dataset in both the brain (A) and blood (B) are strongly preserved (Zsummary > 10) in the original dataset.


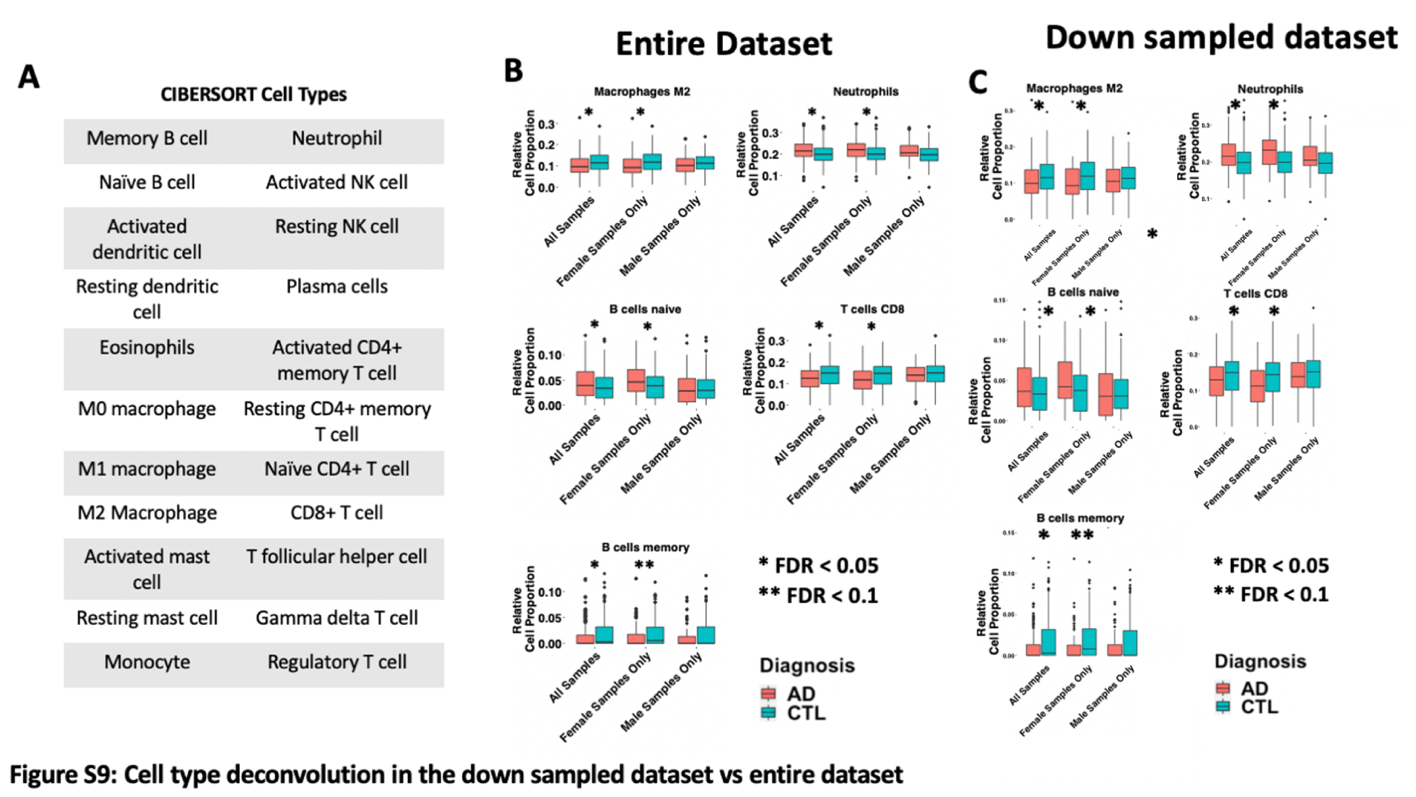
**Figure S10: Cell type deconvolution in the down sampled dataset vs entire dataset**

Description: To assess whether our cell type deconvolution results are replicated in the down sampled dataset, we selected the same iteration described previously and computed cell type proportions using CIBERSORT, similar to the original manuscript. Results are presented for both the entire dataset (B) and down sampled dataset (C). In the down sampled dataset, we observed that levels of M2 macrophages, neutrophils, naïve B cells, CD8 T cells, memory B cells were significantly different between AD cases and controls among females (p<0.05, C). Upon pooling both male and female samples we similarly observed dysregulation in M2 macrophages, neutrophils, naïve B cells, CD8 T cells, memory B cells. We did not observe dysregulation in any of the CIBERSORT cell types (A) among male samples (C). These results in pooled male + female samples, female samples only, and male samples only are consistent with the cell type changes we observed in the entire dataset (B).


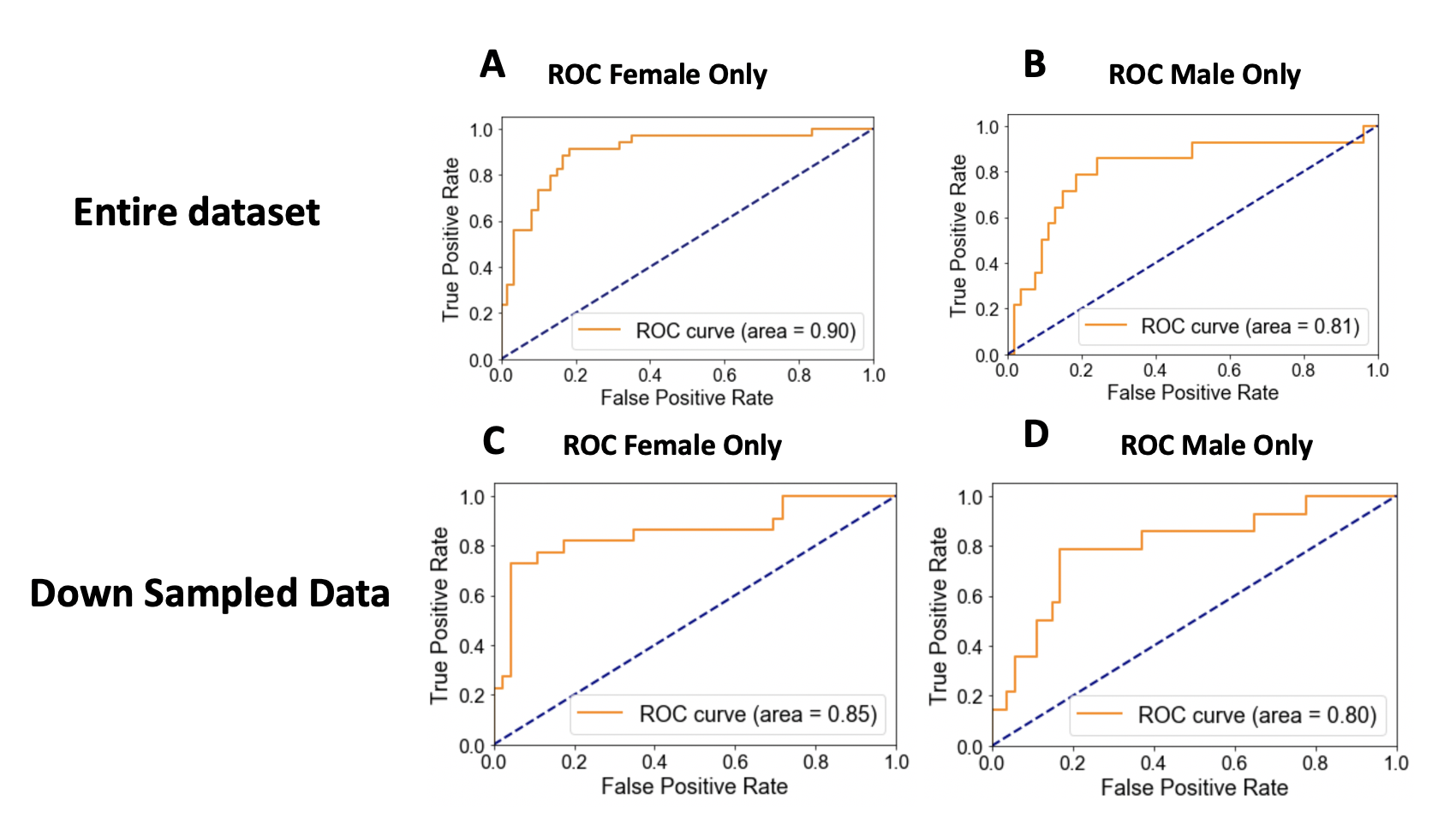


**Figure S11: SVM Classifier performance in down sampled dataset vs entire dataset**

Description: To assess whether the performance of a linear support vector machine (SVM) model with l1 regularization used to classify AD cases and controls based on blood gene expression data was different in the down sampled data compared to the entire dataset, we created receiver operating characteristic (ROC) curves depicting performance of each linear SVM model on a test set composed of 25% of samples. Features include gene expression data obtained via meta-analysis, age, sex, education, and APOE ε4 status. Models were fit for female samples only (A,C), and male samples only (B,D). While we did not down sample the male dataset, the performance in the male dataset was slightly different compared to the original manuscript (AUROC = 0.80 vs AUROC = 0.81 in the original dataset). This difference can be ascribed to using a random seed when training the SVM. In the down sampled dataset, consistent with our original claims (A,B), we observed a higher AUROC in a model trained on female samples (AUROC = 0.85; C) compared to a model trained on male samples (AUROC = 0.80; D). Overall, these results suggest that performance differences in male and female samples are not strongly driven by sample size differences.
